# Donor–acceptor Stenhouse adduct functionalised polymer microspheres†

**DOI:** 10.1039/d2py01591a

**Published:** 2023-02-28

**Authors:** Justus P. Wesseler, Grant M. Cameron, Peter A. G. Cormack, Nico Bruns

**Affiliations:** a WestCHEM, Department of Pure and Applied Chemistry, University of Strathclyde Thomas Graham Building 295 Cathedral Street Glasgow G1 1XL Scotland UK peter.cormack@strath.ac.uk; b Department of Chemistry, Technical University of Darmstadt Alarich-Weiss-Straße 4 64287 Darmstadt Germany nico.bruns@tu-darmstadt.de

## Abstract

Polymers that carry donor–acceptor Stenhouse adducts (DASAs) are a very relevant class of light-responsive materials. Capable of undergoing reversible, photoinduced isomerisations under irradiation with visible light, DASAs allow for on-demand property changes to be performed in a non-invasive fashion. Applications include photothermal actuation, wavelength-selective biocatalysis, molecular capture and lithography. Typically, such functional materials incorporate DASAs either as dopants or as pendent functional groups on linear polymer chains. By contrast, the covalent incorporation of DASAs into crosslinked polymer networks is under-explored. Herein, we report DASA-functionalised crosslinked styrene–divinylbenzene-based polymer microspheres and investigate their light-induced property changes. This presents the opportunity to expand DASA-material applications into microflow assays, polymer-supported reactions and separation science. Poly(divinylbenzene-*co*-4-vinylbenzyl chloride-*co*-styrene) microspheres were prepared by precipitation polymerisation and functionalised *via* post-polymerisation chemical modification reactions with 3^rd^ generation trifluoromethyl-pyrazolone DASAs to varying extents. The DASA content was verified *via*^19^F solid-state NMR (ssNMR), and DASA switching timescales were probed by integrated sphere UV-Vis spectroscopy. Irradiation of DASA functionalised microspheres led to significant changes in their properties, notably improving their swelling in organic and aqueous environments, dispersibility in water and increasing mean particle size. This work sets the stage for future developments of light-responsive polymer supports in solid-phase extraction or phase transfer catalysis.

## Abstract 2

DASA functionalised polymer microspheres as light-responsive species modulate polymer–solvent interactions.

## Graphical abstract

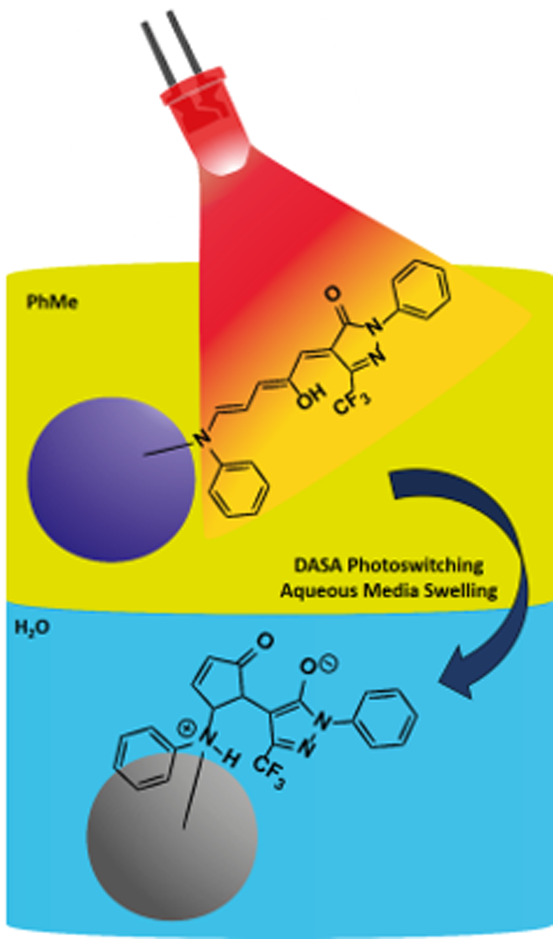

## Introduction

**Scheme 1:**
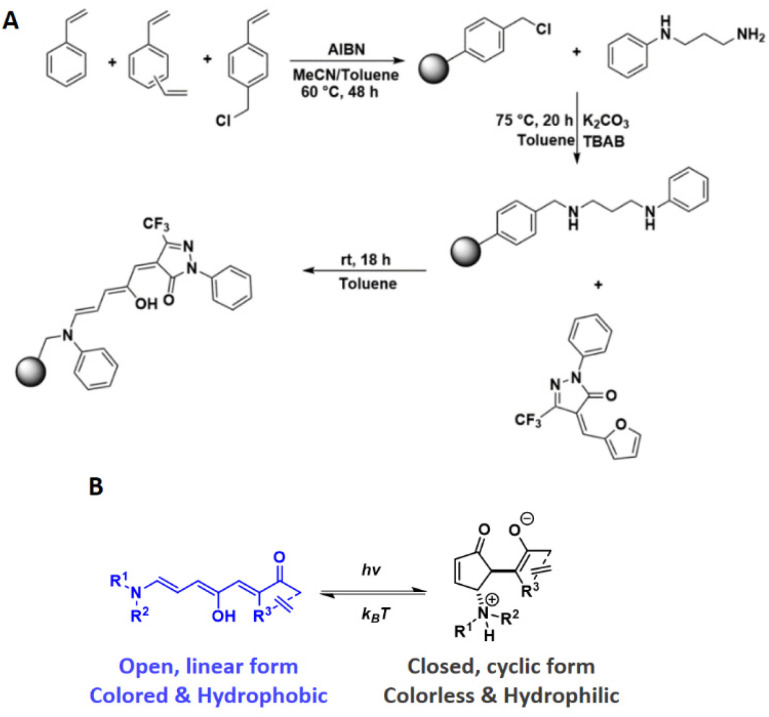
(A) Reaction scheme outlining the generation of crosslinked of poly(DVB-*co*-VBC-*co*-styrene) microspheres through precipitation polymerisation followed by post-polymerisation functionalisation steps to generate the pendant DASA groups. (B) Structures and properties of DASAs in their open and closed form.

Polymer microspheres have received considerable interest as a materials platform due to their advantageous characteristics, such as their ease of preparation, tuneable size and porosity, broad functionalisation chemistry and facile recovery after use.^1–3^ Consequently, they have shown potential in areas such as biomedical applications (*e.g.*, embolization therapy, drug delivery, affinity bioseparators),^4,5^ catalysis,^6,7^ cell micropatterning,^8^ and photonic crystal films.^9^ Concomitantly, the progress in stimuli-responsive polymers, which allow autonomous modulation over a polymer's physical and/or chemical properties, has revolutionised polymer applications. Such stimuli include light,^10^ mechanical force,^11^ temperature,^12^ pH,^13^ electric current^14^ and redox potential.^15^ Light as a stimulus is particularly attractive owing to its non-invasive nature and because it can afford spatiotemporal control over the material's properties. Imparting light-responsiveness onto microspheres has been realised through functionalisation with photochromes such as azobenzenes and spiropyrans, succeeding in modulation of molecular imprinting.^16,17^ Donor–Acceptor Stenhouse Adducts (DASAs) are visible light responsive molecules that have enjoyed substantial interest for the development of new light-responsive materials since their discovery in 2014.^18–21^ DASA development originates from structural and synthetic modifications of their predecessors, Stenhouse salts, which are named after the Scottish chemist, John Stenhouse, who first reported their formation in 1850.^22–24^ Stenhouse salts are obtained by the reaction of furfural with two equivalents of a secondary amine under acidic conditions, to give an intensely coloured triene species. The linear forms of Stenhouse salts were shown to undergo a thermal 4π-electrocyclisation, yielding colourless cyclopentenone species. DASAs comprise a donor group (an aliphatic or aromatic amine) and an acceptor group (a cyclic carbon acid species), linked by a triene chain (Scheme 1B). This “push–pull” molecular system between the amine donor and carbon acid acceptor has been subjected to intensive theoretical investigations^21,25–31^ and synthetic efforts.^18–20,32–34^ Structural changes to the donor and acceptor species enabled modular control over DASA properties, such as switching kinetics, maximum absorption wavelength, thermal equilibria, and solvent compatibility. Activated by irradiation with light spanning the visible to near-infrared wavelength range, the coloured linear triene species undergoes a multistep photothermal process, finally adopting its colourless cyclic form.^21^ This process is thermally reversible in the dark, allow recovery of the coloured starting isomer. This negative photochromism (photoswitching leads to bleaching), allows light to penetrate DASA-functionalised materials efficiently. Additionally, photoswitching is accompanied by changes in hydrophilicity,^19,35,36^ molecular volume^19^ and excitation of DASAs has been shown to result in substantial photothermal heating.^37–39^

Several groups rapidly realised the potential of DASAs to yield light-responsive materials and nanostructures, achieved through the covalent attachment of DASAs to polymers.^34,40–46^ However, the reversible switching process of DASAs is complicated when polymer-bound, as it is additionally dependent on the host polymer's properties. When solvated, DASA–polymers exhibit significantly improved switching behaviour compared to dry, solid-state conditions. In the solid state, effective and reversible switching of polymer-bound DASAs is dependent on the polymer's glass transition temperature (*T*_g_).^41,47^ When held at or above the host polymer's *T*_g_, DASA switching kinetics markedly improve.^38,41,46^ Studies by Sroda *et al*. have also shown that the level of DASA functionalisation itself affects *T*_g_ and elastic modulus of the host polymer,^38^ while Yap *et al*. demonstrated polymer chain-length dependence on DASA switching performance, with shorter chains (of ∼20 monomer units) showing faster switching compared to longer chain-lengths (of ∼100 monomer units).^43^

Despite DASA–polymer interactions being underexplored, the property changes associated with DASA photoswitching has led to the development of new light-responsive materials in the fields of targeted drug delivery,^35,48^ wavelength-selective polymersome biocatalysis,^42,49^ photolithography,^50^ 3D printing,^50,51^ encryption,^52^ surface wettability,^36,53^ detection of toxic chemical warfare agents^54^ and photothermal actuation.^37,38^ While the types of materials into which DASAs have been incorporated are diverse, including inorganic nanoparticles^53,55^ and as dopants in crosslinked polyurethane networks,^56^ covalently-bound DASAs in crosslinked polymer networks have only recently been explored.^51^ We therefore sought to expand the applicability of DASAs in crosslinked polymer microspheres. Herein, we report the synthesis and study the properties of DASA-functionalised styrene–divinylbenzene microspheres with different levels of DASA functionalisation. Precipitation polymerisation (PP) was used to yield micron-sized spherical particles,^57,58^ which have been used previously in a number of different areas, including binding assays,^59^ disease detection,^60^ separation science,^61^ and polymer-supported synthesis.^62^ To the best of our knowledge, this is the first example of DASA functionalised crosslinked polymer microspheres (DMs). The presence of the DASAs in the microsphere network is shown to yield substantial changes in the network's properties in response to irradiation with visible light.

## Experimental

### Materials

Copper(i) chloride (Sigma Aldrich, 99%), iodobenzene (Sigma Aldrich, 98%), 1,3-diaminopropane (Sigma Aldrich, 99%), phenylhydrazine (Sigma Aldrich, 97%), ethyl 4,4,4-trifluoroacetoacetate (Sigma Aldrich, 99%), glacial acetic acid (Sigma Aldrich, 99%), acetonitrile (Sigma Aldrich, 99.8%), toluene (Sigma Aldrich, 99%) and, unless otherwise specified, all other reagents were used as received without further purification. Azobisisobutyronitrile (AIBN, Sigma Aldrich, 98%) was recrystallized from cold acetone. Divinylbenzene 80% (DVB-80, Sigma Aldrich), 4-vinylbenzyl chloride (VBC, Sigma Aldrich, 90%), and styrene (Alfa Aesar, 99%) were all purified by filtering through an aluminium oxide (neutral, Brockmann type I) column prior to use. Furfural was distilled (90 °C, 90 mbar) before use and stored at −20 °C.

#### Preparation of *N*-phenylpropane-1,3-diamine and 2-phenyl-5-(trifluoromethyl)-2,4-dihydro-3*H*-pyrazol-3-one

Full details of the synthesis of *N*-phenylpropane-1,3-diamine and 2-phenyl-5-(trifluoromethyl)-2,4-dihydro-3*H*-pyrazol-3-one are described in the ESI.†

#### General precipitation polymerisation procedure for the synthesis of poly(DVB-*co*-VBC-*co*-styrene) microspheres

Each batch of polymer microspheres kept the amount of DVB-80 in the feed identical and varied the ratio of VBC to styrene to maintain the same nominal crosslink ratio across all polymers while allowing the DASA loading to be varied systematically. The ratio of monovinyl monomer (*i.e.*, the combined amount of VBC and styrene) to crosslinker (DVB-80) was kept fixed at 75 : 25 mol/mol, with the ratio VBC to styrene being varied. The specific quantities of reagents used for the synthesis of each polymer are described in the ESI.† The polymers were prepared using the general procedure which follows. A glass Duran bottle was charged with acetonitrile (375 mL) and toluene (125 mL), and the mixture of solvents placed in an ultrasonic bath to degas for approximately 15 min. DVB-80, VBC and styrene were then added to the reaction flask (10 g of monomer in total), and the homogeneous monomer solution was purged with N_2_ gas for approximately 10–15 min. Once the N_2_ gas purging was complete, AIBN (2 mol% relative to vinyl groups) was added and the bottle sealed and placed on an IBI Scientific low-profile roller housed inside a Stuart Scientific SI160D incubator. The reaction vessel was rolled gently about its long axis at an approximate rotation speed of 5 rpm; the incubator temperature was set to 60 °C and the polymerisation allowed to proceed for 48 h. After this time, the milky suspension of polymer microspheres was isolated by vacuum filtration using a 0.45 μm nylon membrane filter (Sartorius). The microspheres were washed with acetonitrile and acetone (100 mL each). The polymer microspheres were dried overnight *in vacuo* (approximately 10 mbar) at 40 °C to yield white, free-flowing powders in yields of around 1.5 g (15%).

#### Post-polymerisation chemical functionalisation of poly(DVB-*co*-VBC-*co*-styrene) microspheres

The first functionalisation step, which involved installing the DASA-donor *N*-phenylpropane-1,3-diamine into the microspheres, followed a general procedure which is given below for A1.

Poly(DVB-*co*-VBC-*co*-styrene) microspheres (0.5 g, 0.48 mmol of calculated chloromethyl functional groups) were added to a three-necked flask equipped with an overhead stirrer and condenser. Toluene (50 mL) was added to the dry polymer and the resulting suspension was left stirring at 100 rpm for approximately 2 h to allow the polymer microspheres to disperse and swell. Afterwards, *N*-phenylpropane-1,3-diamine (0.10 g, 0.66 mmol) was added to the reaction flask along with potassium carbonate (0.14 g, 1.0 mmol) and tetrabutylammonium bromide (TBAB) (0.04 g, 0.12 mmol). The reaction vessel was heated to 75 °C, the stirring rate increased to 150 rpm and the mixture left to stir for 20 h. After this time, the suspension of polymer microspheres was filtered by vacuum through a 0.45 μm nylon membrane filter (Sartorius) and the microspheres were washed with acetonitrile, acetone, 0.1 M HCl and deionised (DI) water (50 mL each). The isolated polymer was then dried overnight *in vacuo* (approx. 10 mbar) at 40 °C to yield off-yellow powder A1 (0.45 g, 90%).

#### Polymer-bound DASA preparation

The second post-polymerisation chemical functionalisation procedure involved the formation of pendant DASA groups through the furan ring-opening reaction of the trifluoromethyl pyrazolone furan adduct (CF3PFA, experimental procedure S1†) with the amine-functionalised polymer particles. This is described below for D1.

*N*-Phenylpropane-1,3-diamine-functionalised polymer microspheres (0.25 g, 0.24 mmol of calculated secondary amine groups) and toluene (50 mL) were added to a round-bottomed flask equipped with an overhead stirrer. The mixture was stirred for approximately 2 h at a rate of 100 rpm to allow the polymer microspheres to disperse and swell in the reaction solvent. CF3PFA (82 mg, 0.27 mmol) was then added to the reaction flask and the deep orange coloured mixture was left stirring at room temperature overnight at a speed of 100 rpm. The final dark blue mixture was filtered through a 0.45 μm nylon membrane filter (Sartorius) and the solids washed with acetonitrile and acetone before being dried overnight *in vacuo* (approximately 10 mbar) at 40 °C to yield a blue-coloured polymer product (0.26 g, 97%).

### Characterisation methods

#### Scanning electron microscopy (SEM)

Polymer microspheres were coated prior to analysis using a Polaron SC500A sputter coater equipped with a gold–palladium disc. The samples were coated for 8 min (4 min of direct coating and a further 4 min at an angle of 45°). Coated samples were then placed inside a Cambridge Instruments Stereoscan 90 scanning electron microscope and analysed using a beam voltage of 25 kV. The SEM images were analysed using ImageJ software. The diameters of one hundred beads were measured, and the corresponding mean, standard deviation (SD), and coefficient of variation (CV) were calculated.

#### Elemental microanalysis

The carbon, hydrogen and nitrogen (CHN) analysis of polymer samples was carried out by the University of Strathclyde's Elemental Microanalysis Service. 1–3 mg of sample was weighed into a tin foil pan and the pan folded closed before being placed inside a PerkinElmer Series II analyser. The polymer samples were burned at 1800 °C in a pure oxygen environment.

#### UV-Vis analysis

UV-Vis measurements were performed using a Shimadzu UV-2600 UV-Vis spectrophotometer with integrating sphere for diffuse reflectance measurements. For diffuse reflectance measurements, DMs were dispersed in toluene at a DM concentration of 0.15 mg mL^−1^. The microspheres were stirred for 24 h in the dark prior to data acquisition to allow for sufficient swelling of the spheres and equilibration of the DASAs. During analysis, DM dispersions were transferred into quartz cells with a path length of 10 mm. Photoswitching of DASAs was achieved by irradiating with a ThorLabs LED array (LIU630A, 630 nm), fastened *via* a clamp approximately 5 mm away from the cuvette perpendicular to the beam of the UV-vis spectrometer. The intensity of the incident light in this setup was measured to be 1.5 mW cm^−2^. Monitoring the thermal reversion in the dark was done by leaving the cuvette in the spectrophotometer. Measurements were taken at regular time intervals. The cuvette was regularly agitated by hand to prevent microsphere sedimentation. The raw data was smoothed using a Savitzky–Golay filter and normalised using Origin software before the plotting of kinetics.

#### Solid-state NMR (ssNMR)

Solid-state NMR analysis of the DMs was carried out by the NMR facility at Durham University. The spectra were recorded using a Bruker Avance III HD spectrometer operating at 376.51 MHz for fluorine and using a 3.2 mm rotor MAS probe. Spectra were obtained at a spin-rate of 20 kHz using a rotor-synchronised spin echo allowing enough relaxation delay to ensure full relaxation. Spectral referencing was done with respect to CFCl_3_ and by setting the signal from 50% CF_3_COOH in H_2_O to −76.54 ppm. To determine the DASA content within the microspheres, 4,4′-difluorobenzophenone (DFB) was used as an external standard. *T*_1_ for DFB was found to be 473 s. Obtaining a fully relaxed spectrum was achieved using a transient wait of 2375 s (5 × *T*_1_) and 1 repetition. For the DMs, a recycle delay of 5 s was sufficient, with the number of repetitions ranging from 256–512 to improve sensitivity. To account for the varying repetitions, raw integral values were corrected for the number of repetitions done and the mass of each sample (Table S4†), to yield a value for integral per mg. These values were then normalised with the integral value per mg for DFB being equal to 100, allowing for quantification of DASA functionalisation *via* the CF_3_ group integrals (full calculations found in ESI†).

#### Nitrogen sorption analysis

Nitrogen sorption analysis was carried out using a Micromeritics ASAP 2020 analyser. A minimum of 0.1 g of sample was added to a glass sample tube in the instrument and degassed initially for 2 h at 50 °C and then for a further 20 h at 70 °C, both under high vacuum (reduced pressure of 35 μmHg). Once completed, the analysis step of the procedure was carried out, which involved the dosing of the sample tube with nitrogen gas of known partial pressures. A total of 70 data points were taken to form the isotherm for the sample being run. The isotherm data is then reported along with values for specific surface area (SSA), specific pore volume and mean pore size.

#### Differential scanning calorimetry

Differential scanning calorimetry was performed using a TA Instruments Q20. 5 mg of polymer sample was weighed and hermetically sealed in an aluminium DSC pan and placed in the instrument. Samples were measured under nitrogen flow from room temperature to 225 °C at 10 °C min^−1^.

#### Swelling studies

For the photoswitching, an IKEA Forsa table lamp with a RYET 400 lm LED was used to irradiate the DM dispersions in toluene for 24 h. Upon completion, the vial was removed from under the lamp and the DMs were isolated by filtration through a 0.45 μm nylon membrane filter (Sartorius), washed with acetone, dried *in vacuo* (approximately 10 mbar) at 40 °C overnight, and re-suspended in vials containing water and toluene for swelling studies. DMs that were not irradiated were dispersed directly in vials in either toluene or water and left to swell. The vials were placed on a IBI scientific low-profile roller or a Labnet Gyromini nutation mixer (depending on the size of the vial). The swelling was measured over 24 h at regular time intervals. DMs were collected for swelling measurement by centrifugation, discarding the supernatant and filtering the DMs by vacuum on a 0.45 μm nylon membrane filter (Sartorius). Following weighing of the DMs, they were redispersed into the solvent.

#### DM partitioning in two-phase systems

Investigations into the partitioning behaviour of DMs were carried out by dispersing a sample of dry polymer in a sample vial using a suitable solvent (toluene/chloroform) at a concentration of 1 mg mL^−1^. After allowing the DMs to swell completely overnight, an equivalent volume of water was added to the vial, to give a two-phase system, together with a magnetic stirrer bar. Photoswitching was carried out by exposure of the sample vial to a white-light source (IKEA Forsa table lamp fitted with RYET 400 lm LED) whilst under stirring. Once irradiation was complete, the magnetic stirrer bar was removed and further agitation was carried out by hand during the investigation of the partitioning behaviour.

#### SEM investigation of size change of DMs upon photoswitching

Investigation into the effect of photoswitching on the DMs’ particle size was carried out by first analysing open-form DASA DMs by SEM. Following photoswitching using the procedure described above (*i.e.* dispersion in solvent, irradiation under desk lamp, *etc*.) and isolation of dry DMs, SEM analysis was repeated and the data acquired before and after photoswitching compared.

## Results and discussion

### Synthesis of DASA-functionalized polymer microspheres

The preparation of the DASA-functionalised polymer microspheres was achieved in a sequential approach involving a precipitation polymerisation followed by polymer-analogous reactions (Scheme 1). Poly(DVB-*co*-VBC-*co*-styrene) polymer microspheres with varying VBC contents were prepared by precipitation polymerisation, the chloromethyl groups providing the functional handle for post-polymerisation chemical modification reactions (Scheme 1A).

Installation of the DASA-donor on the microspheres and subsequent reaction with the CF_3_-pyrazolone furan adduct then created the 3^rd^ generation DASA groups on the microspheres. The aniline donor and 2-phenyl-5-(trifluoromethyl)-pyrazolone acceptor (CF3Pyra) were chosen for the rapid switching kinetics of the resulting DASA, both for the forward light-induced photoisomerisation and the thermal reversion process, its favourable equilibria (being close to 100% open form at room temperature) and its ease of synthesis.^20^ The nominal crosslink ratio used for all polymer syntheses was 25 wt%, to allow microspheres to form and precipitate during polymerisation whilst also allowing for swelling of the microspheres in compatible solvents. Having swellable microspheres was expected to facilitate the polymer-analogous reactions and aid DASA switching. The yields, particle size and particle size distribution of the polymer microspheres are presented in Table 1.

**Table:** Polymer microsphere yields and particle size. P, A and D denote the functionalisation stage of the polymer, *e.g.*, chloromethyl-containing polymer obtained after precipitation polymerisation (P), amine-functionalised (A) or DASA-functionalised polymer microspheres (D) with 1, 2, 3 distinguishing the three different variations in functional group loading levels based on the initial monomer feed

Polymer		Polymer yield		SEM analysis	
		(g)	(%)^a^	*D* (μm)	CV (μm)
P1	10 mol% VBC precursor^b^	0.89	9	4.41	1.01
A1	Amine-functionalised P1	0.45	90	4.25	0.94
D1	DASA-functionalised A1	0.26	97	4.31	0.91
P2	30 mol% VBC precursor^b^	1.35	13	5.52	1.44
A2	Amine-functionalised P2	0.52	98	5.72	1.26
D2	DASA-functionalised A2	0.26	105	5.09	1.22
P3	50 mol% VBC precursor^b^	1.59	15	4.81	1.16
A3	Amine-functionalised P3	0.53	106	5.50	0.94
D3	DASA-functionalised A3	0.27	110	5.20	1.09

a Yields for P1–3 were calculated by comparison of the mass of the isolated polymer to that of monomer and initiator, whilst the yields of A1–3 and D1–3 were calculated by comparison of the mass of product to the mass of polymer added as a starting material.

b Mol% refers to the amount of chloromethyl functional groups present in the initial polymer microspheres; these were then used for functionalisation reactions.

By varying the ratio of styrene to VBC in the monomer feeds, DASA-functionalised polymer microspheres with controllable DASA functionalisation levels could be obtained. The level of VBC monomer used in the monomer feeds was 10, 30 and 50 mol% relative to total monomer. Yields of polymer microspheres were 9–15% (Table 1). While low yields of microparticles are typical for the precipitation polymerisation of these monomers under such dilute conditions (monomer concentration 2% w/v),^58^ this was not an impediment for the present work because the synthesis of microspheres follows an easy and scalable protocol and because the microspheres had the desired chemical composition and physical form. Analysis of the microspheres by SEM showed that they comprised discrete spherical particles, with mean particle diameters between 4 and 6 μm and coefficient of variation (CV) ∼20% for all samples (Fig. S8† and Table 1).

FT-IR spectroscopic analysis of the P1–3 microspheres (for all FT-IR spectra, see ESI 8†) provided evidence for the presence of all three monomers in the final products. The signal at 1265 cm^−1^ is diagnostic for chloromethyl groups derived from VBC, and the intensity of this signal increases in the order P1 to P2 to P3 as the level of VBC in the monomer feed is increased. In addition, all poly(DVB-*co*-VBC-*co*-styrene) microspheres gave CHN values that were almost identical to their calculated expected percentages (Table 2). Assuming statistical incorporation of all three monomers, the chemical composition of the polymers mirrors that of the monomer feeds. In other words, there is no evidence of compositional drift for this set of three polymer microspheres.

**Table:** Elemental microanalysis data for polymer microspheres before and after chemical treatment with *N*-phenylpropane-1,3-diamine

Polymer	Elemental microanalysis^a^		
	C (%)	H (%)	N (%)
P1	88.0 (88.3)	7.6 (7.7)	0.6 (0.6)
A1	88.5 (88.4)	7.8 (8.1)	1.4 (3.5)
P2	83.5 (82.8)	7.1 (7.4)	0.5 (0.5)
A2	85.5 (86.2)	7.7 (8.1)	3.1 (5.5)
P3	80.8 (79.5)	6.9 (7.2)	0.5 (0.5)
A3	84.5 (83.1)	7.6 (8.5)	4.0 (8.0)

a Values in parenthesis denote the calculated expected CHN values.

Functionalisation of the VBC-containing polymer microspheres with *N*-phenylpropane-1,3-diamine, using potassium carbonate as a base and phase-transfer catalyst TBAB at 75 °C overnight, yielded off-white/yellow powders. Empirical evidence in support of successful functionalisation of the polymer networks with the amine donor was obtained by FT-IR spectroscopy and CHN elemental microanalysis. The FT-IR analyses of all samples showed a significant reduction in the intensity of the VBC-derived chloromethyl signal at 1265 cm^−1^. Further evidence for successful functionalisation comes from the increased percentage nitrogen content (N%) of the polymer microspheres after functionalisation with *N*-phenylpropane-1,3-diamine, compared to their non-functionalised precursors (Table 2). The nitrogen contents of the functionalised polymer microspheres fell slightly short of the calculated theoretical values for full consumption of chloromethyl groups. This shows that not all of the VBC residues reacted with *N*-phenylpropane-1,3-diamine and, indeed, the chloromethyl-derived signal in the FT-IR spectra did not disappear completely upon going from precursor polymer to functionalised derivative. Converting these %*N* values to amine loading levels gives calculated values of 0.30, 1.40 and 1.60 mmol g^−1^ for A1, A2 and A3, respectively, which can also be expressed in terms of the percentage of functionalised monomer units (3.7 mol%, 15.9 mol% and 25 mol% for A1, A2 and A3, respectively).

The conversion of pendent VBC groups to amine groups was low but not unexpected for heterogeneous reactions such as these. It must be kept in mind that for a successful polymer-analogous reaction on these polymers, the reaction solvent must wet/swell the polymer microspheres and dissolve the small molecule amine and base. In this case, non-polar organic solvents wet/dissolve the polymer and amine, but not the base. Furthermore, functional molecules must penetrate the polymer network and access the reactive sites, and this can be limited by the crosslinking.^63^ Nonetheless, the level of incorporation of the DASA-donor group into the polymer microspheres was deemed sufficient to proceed with the second step of the DASA synthesis to yield novel photochrome-functionalised polymer microspheres.

DASA-functionalised polymer microspheres were prepared by adding CF3PFA to a pre-swollen dispersion of the amine-functionalised polymer microspheres in toluene. Successful formation of DASA was evident by the appearance of a deep blue colour. To provide insight into the final degree of functionalisation of the DMs, ^19^F MAS ssNMR was conducted after filtering off residual free furan adduct. Using 4,4′-difluorobenzophenone as an external standard, the signal of the pyrazolone CF_3_ group at −62 ppm was used to determine the degree of DASA functionalisation (Fig. S1–S4†). Conversions of the pendent aniline-derived amine groups to the DASA were calculated as 41%, 21% and 59% for D1, D2, D3, respectively (Table 3), which equates to DASA loading levels of 0.13, 0.32 and 0.95 mmol g^−1^ (or 1.5 mol%, 3.7 mol%, 14.8 mol%) for D1, D2 and D3, respectively. Incomplete conversion of the pendent aniline groups to DASA is most likely due to the limited accessibility of the furan adducts to some of the polymer-bound amine groups.

Summary of functionalisation steps for all microspheres

**Table:** 

Polymer	Aniline functional units^a^ (mol%)	VBC conversion
A1	3.7	33%
A2	15.9	51%
A3	25.0	50%

**Table:** 

	DASA functional units^b^ (mol%)	Aniline conversion
D1	1.5	41%
D2	3.7	21%
D3	14.8	59%

a Calculated from elemental microanalysis data.

b Calculated from ^19^F MAS ssNMR spectroscopic data.

While we assume that the reaction of CF3PFA to form the DASA proceeds through the nucleophilic attack of the aniline moiety, the benzylic amine arising from the functionalisation of the microspheres with the donor could also react to form DASAs. One way to assess which amine formed the DASAs is to examine their UV-vis spectra. Previous work by Read de Alaniz and co-workers indicates that DASAs with trifluoromethyl pyrazolone acceptors and alkyl amine donors have a *λ*_max_ of 600 nm in toluene, while switching to an aromatic indoline donor yields a DASA with *λ*_max_ = 655 nm in toluene.^20^ However, crucial to this large bathochromic shift with the indoline donor is the 5-membered ring ensuring planarity of the aromatic system. This is not the case for aniline donors, and leads to an out-of-plane twist for the aromatic ring, diminishing the effect of extending the conjugation in the linear triene form.^32^ Indeed, the bathochromic shift between DASAs bearing alkyl amine donors and aniline donors with the same acceptor is much smaller (*ca.* 12 nm).^32,64^ The absorption bands of the DMs are approximately centred on 600 nm, have one maximum only and no shoulder at higher wavelengths, which indicates that only one type of DASA formed. However, as the absorption bands are relatively broad, it cannot be excluded that a small quantity of a second type of DASA formed, and the UV-Vis spectra do not allow one to unequivocally determine which nitrogen centre formed the DASAs. However, ^19^F ssNMR and photoswitching behaviour would suggest that the aniline species is the preferred donor for DASA formation in our case. One main species is observed in the ^19^F ssNMR spectrum (Fig. S2–S4†), and this DASA is clearly able to undergo light-induced ring-closure. Therefore, this DASA must be aniline-based as the combination of an alkyl amine donor with trifluoropyrazolone acceptor was shown previously to be unable to cyclise in toluene.^20^ While the benzylic amine would be the stronger nucleophile, CF3PFA is likely experiencing steric hindrance in approaching it, due to its proximity to the main styryl network.

**Fig. 1:**
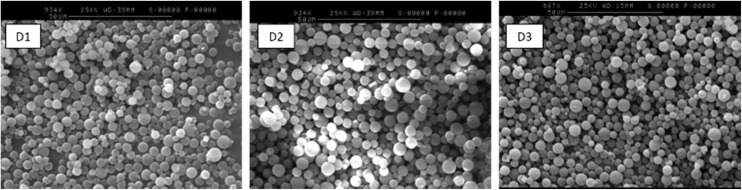
SEM images of DASA polymers D1–3 (scale bar = 50 μm).

An important observation made throughout the functionalisation steps was that there were no obvious changes to the morphology or size of the polymer microspheres. SEM analysis of A1–3 (Fig. S9†) and D1–3 (Fig. 1) showed discrete particles of similar size across each series.

In summary of the synthetic work, it was possible to prepare crosslinked polymer microspheres decorated with DASAs, and to control the level of DASA functionalisation. Having a series of DMs with varying functionalisation levels in hand, we then investigated their photoswitching behaviour.

### DASA switching behaviour in microspheres

**Fig. 2:**
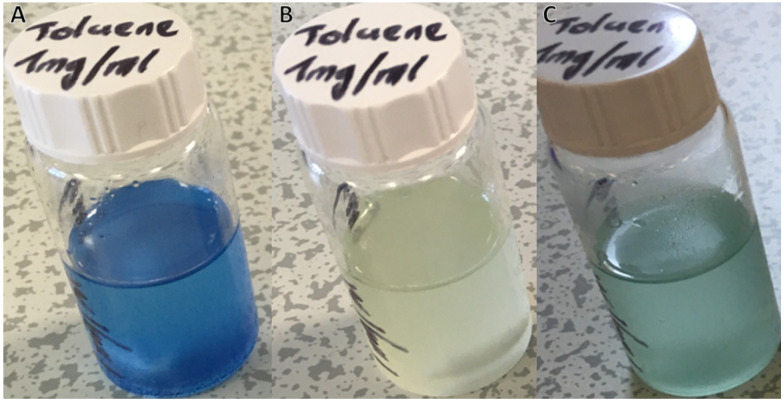
Photos of DASA-functionalised polymer microspheres (D1) in toluene. (A) After being left to swell and equilibrate in the dark for 24 h. (B) After 24 h of irradiation with white light. (C) After a further 24 h in the dark at room temperature.

For polymeric DASA systems, switching behaviour is dictated by certain properties of the polymer matrix. *T*_g_, chemical structure of the repeat unit and polymer chain length can all have a noticeable effect on DASA photoswitching.^41,43^ Furthermore, work by Mostafavi *et al*.^65^ and Sinawang *et al*.^40^ demonstrated that irradiation of DASA–polystyrene (PS) systems, where the DASA was either mixed with or covalently bound to linear polymer chains, led to irreversible photoswitching in the solid state. At ‘high’ loading levels (0.08 mass fraction), even non-covalently bound DASAs in PS require extensive irradiation times (+10 h) to realise complete cyclisation of the DASAs.^65^ In the case of crosslinked polymer microspheres, DASAs are either surrounded by a crosslinked polymer network or on the surfaces of the spheres. We therefore assume two possible environments for a DASA to find itself in, and therefore two main types of photoswitching behaviour. Although comparable to many other crosslinked polymer microspheres, the microspheres presented herein have moderate levels of crosslinking (approximately 25 mol% relative to total monomers). The crosslinking level enables the microspheres to form in the first place and wet/swell when in contact with compatible solvents. However, crosslinking also inhibits local segmental motion in the dry state, even at elevated temperatures (no glass transition was observed by DSC, Fig. S11†). DASAs within a microsphere are thus likely to exhibit reduced photoswitching performance, even when the microspheres are in contact with a good swelling solvent. In contrast, any DASAs located at the outer surfaces of the microspheres should have sufficient free volume to behave similarly to DASAs bound to linear polymers in solution, thereby allowing for faster switching and reversibility. To probe DASA photoswitching on DMs, an initial study was conducted. Suspensions of D1 in toluene (1 mg ml^−1^) were prepared and left for 24 h in the dark, to allow DM swelling and DASAs to equilibrate (Fig. 2A). Toluene is a suitable solvent for both microsphere swelling and DASA photoswitching.^20,66^ Thereafter, irradiation of the suspension of D1 in toluene with white light for a period of 24 h led to the initially coloured suspension becoming fully colourless (Fig. 2B). Finally, the D1 suspension was left in the dark for 24 h, whereupon it regained some of its colour (albeit a less vibrant colour compared to its initial state) (Fig. 2C). The sluggish thermal recovery of DASA–polymer conjugates can be overcome by holding the conjugate above its *T*_g_,^38,41^ although this is complicated in this case due to moderately crosslinked polymer microspheres having no *T*_g_. Attempts to improve the thermal recovery by heating the DMs up to 100 °C in the dark did not lead to any further recovery. It should be noted that the stability of 3^rd^ generation DASAs at these high temperatures is questionable, especially given the recent insights that stronger electron withdrawing acceptors are more susceptible to degradative processess.^67^ However, this is mainly an issue in polar, protic solvents and, furthermore, no change in colour was observed in our case, which suggests that degradation/retro reaction to yield free furan adduct did not occur in the present case.

**Fig. 3:**
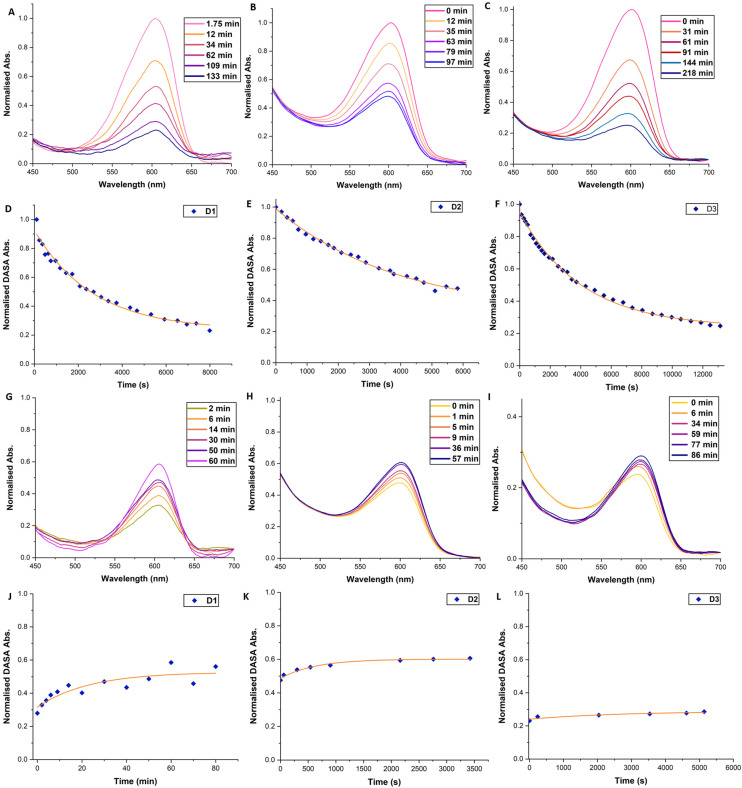
UV-vis spectra and graphs showcasing the change in the DASA absorbance acquired in diffuse reflectance mode. DMs were suspended in toluene (0.15 mg mL^−1^) and left in the dark overnight. Irradiation source: ThorLabs LED array red (630 nm), 1.5 mW cm^−2^ at the sample. Normalised absorbance against time plots were fitted with a mono-exponential decay curve. (A–C) Selected spectra showing decrease in DASA absorbance band for D1–3 during irradiation. (D–F) Normalised absorbance against time plot for D1–3 during irradiation. Orange line shows mono-exponential fit curve. (G–I) Selected spectra showing increase in DASA absorbance band for D1–3 post-irradiation in the dark. (J–L) Normalised absorbance against time plot for D1–3 post-irradiation in the dark. Orange line shows mono-exponential fit curve.

Monitoring of the photoisomerisation behaviour of the DMs by UV-Vis spectroscopy was possible using an integrating sphere UV-Vis spectrophotometer. This set-up allows measurement of the DASAs absorbance band in diffuse reflectance mode, overcoming issues arising from the inherent scattering of microspheres^68^ and the heterogeneous nature of microsphere suspensions (Fig. 3). D1, D2 and D3 suspensions in toluene (0.15 mg mL^−1^) were prepared and left to swell and equilibrate as outlined above. DM suspensions were then transferred to a sealed cuvette and irradiated with a ThorLabs 630 nm LED array (2.4 mW cm^−2^), with incremental scans taken. Once no further obvious reduction in the absorbance of the DASA was observed, the DM suspension was left in the dark to thermally equilibrate, taking further incremental scans. The change in the maximum absorbance wavelength of the DASA over time was plotted and fitted with single exponential decay functions. In the first 2–4 hours, a strong initial decrease in DASA absorbance is observed, which reaches a photothermal stationary state (PTSS) for D1 and D3 and almost for D2 (Fig. 3D–F). During irradiation, the DASA band decreased by 83%, 47% and 70% for D1, D2 and D3, respectively (determined by integrating the area of the DASA absorbance band of the first and final timepoint, Fig. S15†). The DASA concentrations of D1, D2 and D3 in the toluene suspensions were calculated to be 1.95 × 10^−5^ M, 4.80 × 10^−5^ M and 1.42 × 10^−4^ M, respectively. Thermal recovery in the dark plateaued between 40–90 minutes for the various DM compositions (Fig. 3J and K). For D1, D2 and D3, the DASA absorbance band recovery in the dark was 34%, 10%, and 0.1%, respectively. Incomplete bleaching and low thermal recovery were expected for these DASA–polymer systems due to the restrictive polymer environment.^38^ Nonetheless, it was still gratifying to observe first-order switching behaviour for DMs, albeit at timescales much longer than for other DASA–polymer systems. Complete decolourisation after prolonged irradiation (6+ hours) was possible, potentially indicating a portion of DASAs residing in environments where solvent accessibility is restricted and energy barriers for the 4π-electrocyclisation are higher. However, photodegradation under such extensive irradiation periods cannot be ruled out either.

## Effect of photoswitching on the size, porosity and wettability of the polymer microspheres

### DASA-photoswitching modulated microsphere swelling

When lightly crosslinked polymer microspheres come into contact with a compatible solvent, favourable polymer–solvent interactions lead to solvent uptake and swelling of the microspheres. Depending on the level of crosslinking and the polymer–solvent interactions, the microspheres may be able to swell to a number of times their original weight/size.^69^ This feature allows such polymers to be loaded with small molecules and used as carriers for a variety of different applications (*e.g.*, for drug delivery^70^). To probe if the swellability of DMs in aqueous and non-polar organic media could be controlled by DASA photoswitching, DMs were suspended in both water and toluene before and after irradiation (Table 4). To afford DMs with DASAs in their closed state, the DMs were suspended in toluene due to its ideal polymer–solvent interactions and being a good solvent for DASA photoswitching. D2 suspensions were irradiated for 24 hours with white light to achieve complete decolourisation/quantitative closed form hydrophilic DASAs, after which the DMs were filtered, washed and dried before being re-suspended in water and toluene for swelling studies.

**Table:** Swelling results of D2 in toluene and water before and after irradiation with white light for 24 h in toluene

Polymer	Solvent	Initial mass/mg	Swelling time							
			2 h		4 h		6 h		24 h	
			mg	Swelling	mg	Swelling	mg	Swelling	mg	Swelling
D2 open	Toluene	10.2	27.0	165%	30.3	197%	49.7	387%	67.0	557%
D2 open	Water	12.9	16.4	27%	18.3	42%	18.4	43%	19.9	54%
D2 closed	Toluene	2.6	15.7	504%	21.9	742%	38.9	1396%	48.6	1769%
D2 closed	Water	1.5	7.8	420%	9.1	507%	13.8	820%	16.8	1020%

The swellability of non-irradiated (D2 open) and irradiated (D2 closed) DMs was then tested over 24 hours with gentle roller stirring in either toluene or water and measured at regular intervals by increases in their mass (Table 4). Large mass increases in toluene were observed for D2 open along with small water uptake, since the hydrophobic divinylbenzene-based polymer matrix interacts favourably with toluene but not with water. The slight mass increase observed for D2 open in water could be due to water interacting with DASAs groups on the surface of the microspheres,^71^ not entirely shielded by the hydrophobic microsphere network.

Post-photoswitching, a significant increase in the swellability of D2 closed in both solvents was observed. Crucially, the swelling in water was greatly improved. The enhanced swelling of D2 closed in water following irradiation is likely due to closed-form DASAs’ increased hydrophilicity.^15^D2 closed also show improved swelling in toluene. This may arise from the molecular contraction of DASAs adopting their closed-form, which offers more free volume for solvent molecules to penetrate and swell the matrix further.^19,65^ The swelling data demonstrates that DASA photoswitching profoundly modulates solvent penetration into the crosslinked networks, particularly for aqueous solvents.

Furthermore, it was investigated whether photoswitching also changed the mean particle size (Table 5). Dried samples of DMs were analysed by SEM before and after swelling and white light irradiation in toluene. Following 4 h irradiation of DMs in toluene, filtration and drying, clear increases in mean particle size were observed for D2 and D3. The mean diameter of the D1 particles remained within 2% of their original value during DASA photoswitching, suggesting that the concentration of DASAs in these microspheres is simply too low to exert a measurable change to the dry particle size. After 24 hours irradiation, the size exceeds the initial size pre-irradiation for D2 (+0.71 μm) and D3 (+0.41 μm), indicating that the molecular rearrangements of DASA photoswitching disrupts the network sufficiently in the materials with higher DASA loading levels. However, the mean diameter is smaller than after 4 h irradiation, which could arise from the polymer network equilibrating once DASA photoswitching is complete.

**Table:** Particle size of dry DMs before and after DASA photoswitching in toluene. Irradiation was carried out with white light, mean particle size was determined by SEM analysis (images in ESI†)

Polymer	Irradiation time/h	Sphere size	
		*D* (μm)	CV (μm)
D1	0	4.31	0.91
	4	4.40	1.06
	24	4.29	0.90
D2	0	5.09	1.22
	4	6.12	1.41
	24	5.80	0.99
D3	0	5.20	1.10
	4	6.30	1.10
	24	5.61	1.07

### Porosity of the DMs

**Fig. 4:**
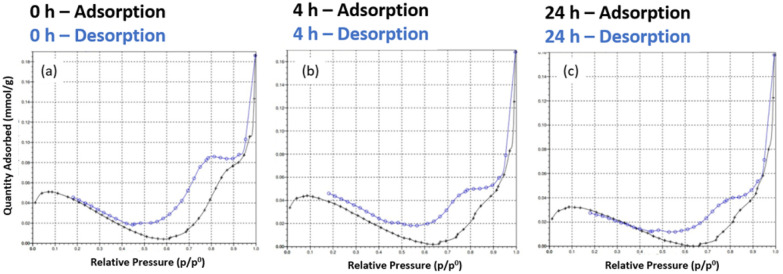
Nitrogen sorption isotherms of D2. (a) In their initial state, (b) after 4 h of white light irradiation, (c) after 24 h of white light irradiation.

Assessment of the porosity of the D2 DMs by nitrogen sorption analysis showed that the microspheres had a specific surface area (SSA) of <5 m^2^ g^−1^, which indicates a generally non-porous solid in the dry state (Fig. 4). The negative slope in the isotherms at partial pressure range 0.1–0.6 indicates the presence of residual moisture and further confirms lack of adsorption/porosity. Moreover, no effect of DASA photoswitching on the dry state SSA of the polymers was observed. Most likely this is because the polymer network is not crosslinked enough to prevent collapse of the solvent-expanded network when the solvent is removed. The rapid increase in adsorption at higher partial pressures and desorption hysteresis observed may indicate a macroporous nature, yet the low overall SSA measured prevents definitive conclusions from being drawn. The changing of isotherms with irradiation time does suggest that the DASA's photoswitching modulates the structural composition of the microspheres, but evidently further investigations are needed to clarify this potential effect. (As a side-note, Barret–Joyner–Halenda pore volume distribution plots showed an increasing contribution of a larger pore with increasing irradiation time, Fig. S12,† but once again the low SSA precludes this data from being meaningful.)

### Photo-modulated dispersibility in water

Recent insights into the ground state of 3^rd^ generation DASAs have shown that, alongside their closed form favouring a zwitterionic species, their open form favours a charge-separated, zwitterionic resonance form.^72^ Nonetheless, conversion of linear DASAs to the cyclic form increases hydrophilicity, with studies confirming cyclic DASAs forming stable complexes with water molecules and changes in water contact angles being observed upon photoswitching.^53,71^ With DMs already demonstrating this hydrophobic/hydrophilic transition through improved polymer swelling when placed in aqueous solutions after switching, it was investigated if DMs could have their aqueous dispersibility tuned by irradiation. Ordinarily, the hydrophobic nature of the DVB-based polymers results in creaming and aggregation of microspheres when they are contacted with water (unless the polymer wettability is improved by hypercrosslinking^3^).

**Fig. 5:**
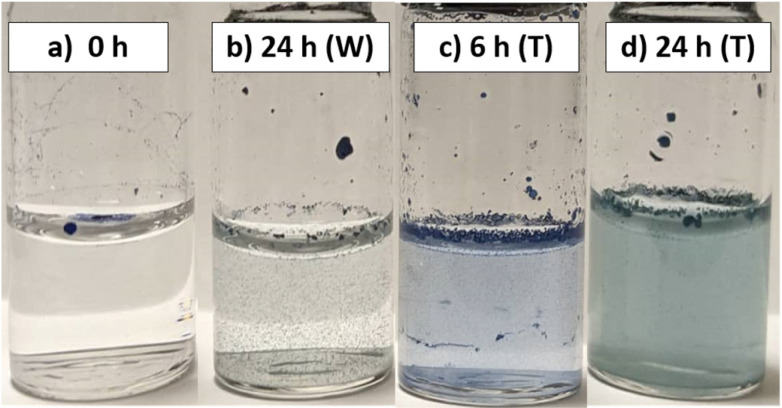
Photographs of aqueous dispersion of DMs as a function of DASA photoswitching. (a) Non-irradiated D2 in water. (b) D2 irradiated for 24 h with white light in water. (c) D2 irradiated for 6 h with white light in toluene, dried and dispersed in water. (d) D2 irradiated for 24 h with white light in toluene, dried and dispersed in water. (All sample concentrations were 1 mg mL^−1^ of D2.)

When D2 was added directly to water, it aggregated at the water–air interface, even upon agitation of the sample vials. Upon extended irradiation (24 h) of the aqueous DM suspension with white light, D2 showed a slightly improved dispersibility in water (Fig. 5). Fig. 5C and D show D2 in water, following prior photoswitching in toluene (1 mg mL^−1^) for 6 and 24 hours, respectively, filtering, drying and re-dispersion in DI water. Improvements in the microsphere–water interactions were observed, indicated by the homogeneous colour of the aqueous phase. Since toluene allows for effective DM swelling, this gives the DASAs a more mobile environment to photoswitch in, and could be considered as a ‘priming’ step for improved water uptake. Following toluene removal and with DASAs locked in their closed form, the DMs were now able to form proper suspensions in water.

Differences in the extent of DASA switching between the 6 h irradiated sample and the 24 h irradiated samples could be distinguished by the naked eye. DMs after 6 h of photoswitching retain a vibrant blue colour, whereas DMs after 24 h of photoswitching have a less intense colour.

**Fig. 6:**
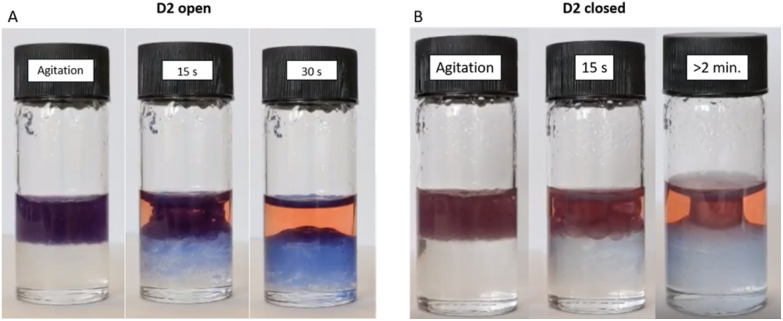
Partitioning of D2 in water (top)/chloroform (bottom) biphasic system. (A) Photographic stills from video: D2 without exposure to intense light source. Progression of D2 after vigorous mixing: full initial dispersion in the aqueous layer, to gradual re-entering into the chloroform layer. (B) Photographic stills from video: D2 which had been irradiated for 24 h in chloroform (1 mg mL^−1^) with white light prior. Progression of D2 after vigorous mixing: full dispersion in the aqueous layer, partial re-entering into the chloroform layer and seemingly stable emulsion formation. The aqueous phase contains Congo Red for clarity.

Since the ability to be dispersed in aqueous media requires pre-emptive photoswitching in toluene, we investigated this phenomenon further in biphasic systems. Two samples of D2 were swollen in chloroform. One sample was photoswitched by irradiation with white light for 24 h, while the other was left in the dark to serve as a control. DI water was then layered on top of the chloroform/DM phase. Upon agitation, both DMs transiently migrate fully into the aqueous layer. The DMs with DASAs in their open form partitioned back into the organic layer within approximately 30 s (Fig. 6A and Video S1†). For DMs with DASAs in their closed form, some partition back into the organic phase was observed, yet a seemingly stable dispersion was visible which persisted when undisturbed (Fig. 6B). Thus, DASA functionalised DVB-based polymer microspheres can be selectively modulated to interact favourably with aqueous environments through photoswitching when swollen in organic solvents prior. Upon agitation, the increased hydrophilicity of the closed form enables the formation of stable dispersions of the DMs in the aqueous phase. This behaviour could make photoswitched DMs interesting candidates for application as heterogeneous polymer supports for metal complex catalysts, organocatalysts and phase-transfer catalysts.

## Conclusion

In conclusion, a reliable method for the preparation of crosslinked polymer microspheres functionalised with DASAs has been established. The DASAs within DVB-PS crosslinked polymer networks are able to undergo photoswitching and thermal recovery in the dark, yet the photoswitching is comparably slow and the thermal back reaction limited by the restrictive environment of the crosslinked network. While a lack of significant reversibility for the DASA switching is disappointing, in the present case it led to substantially improved interactions of crosslinked microspheres with water, with DASA functionalisation levels as little as approximately 3 mol%. The changes in aqueous swellability upon DASA photoswitching lays the foundation for transient phase transfer of the microspheres from an organic solvent to an aqueous phase, so that the DMs could find applications in phase transfer catalysis or for purification or extraction purposes. Optimisation of DASA-functionalised polymer microspheres can be envisioned through changing the constituent monomers to yield more flexible polymer networks to improve thermal reversion of DASAs supported on microspheres.

## Author contributions

J. P. Wesseler: Conceptualisation, investigation, methodology, data curation, formal analysis, writing – original draft, writing – review & editing, visualisation. G. M. Cameron: Conceptualisation, investigation, methodology, data curation, formal analysis, writing – original draft, writing – review & editing. P. A. G. Cormack: Conceptualisation, methodology, resources, funding acquisition, supervision, writing – review & editing. Bruns: Conceptualisation, methodology, resources, funding acquisition, supervision, project administration, writing – review & editing.

## Conflicts of interest

There are no conflicts to declare.

## Supplementary Material

PY-014-D2PY01591A-s001
