# Supplementary material for: Donor–acceptor Stenhouse adduct functionalised polymer microspheres
Source: Polym Chem. 2023 Feb 28;14(13):1456–68. doi: 10.1039/d2py01591a (PMC10043757; doi:10.1039/d2py01591a)
Supplement: PY-014-D2PY01591A-s001 [file PY-014-D2PY01591A-s001.pdf]

Electronic Supplementary Material (ESI) for Polymer Chemistry.
This journal is © The Royal Society of Chemistry 2023

Supplementary Information

Donor-Acceptor Stenhouse Adduct Functionalised Polymer Microspheres

Justus P. Wesseler,[a] Grant M. Cameron,[a] Peter A. G. Cormack[a,*] and Nico Bruns [a,b,*]

[a]Department of Pure and Applied Chemistry, University of Strathclyde, Thomas Graham Building, 295 Cathedral Street, Glasgow, G1 1XL, Scotland, UK.

[b]Department of Chemistry, Technical University of Darmstadt, Alarich-Weiss-Straße 4, 64287 Darmstadt, Germany

SI Experimental Procedures

Table S1. Reagent stoichiometry for synthesis of precursor polymer microspheres.

Polymer	Reagent	Quantity	Amount [mmol]
**P1**	DVB-80	2.82 g	21.6
	Styrene	5.70 g	54.7
	VBC	1.53 g	10.0
	AIBN	0.35 g	2.13
	MeCN	378 mL	-
	Toluene	125 mL	-
**P2**	DVB-80	2.81 g	21.6
	Styrene	3.21 g	30.8
	VBC	4.26 g	27.9
	AIBN	0.31 g	1.9
	MeCN	375 mL	-
	Toluene	125 mL	-
**P3**	DVB-80	2.80 g	21.4
	Styrene	1.52 g	14.6
	VBC	5.73 g	37.5
	AIBN	0.31 g	1.9
	MeCN	375 mL	-
	Toluene	125 mL	-

Table S2. Reagent stoichiometry for synthesis of amine-functionalised microspheres. [a]Calculated from initial monomer feed ratio

Polymer	Reagent	Quantity	Amount [mmol]
**A1**	P1	0.51 g	0.49[a]
	N-phenylpropane-1,3-diamine	0.10 g	0.66
	K_2CO_3	0.14 g	1.01
	TBAB	0.04 g	0.12
	Toluene	50 mL	-
**A2**	P2	0.53 g	1.40[a]
	N-phenylpropane-1,3-diamine	0.20 g	1.33
	K_2CO_3	0.37 g	2.68
	TBAB	0.10 g	0.31
	Toluene	50 mL	-
**A3**	P3	0.51 g	1.85[a]
	N-phenylpropane-1,3-diamine	0.38 g	2.53
	K_2CO_3	0.50 g	3.62
	TBAB	0.13 g	0.40
	Toluene	50 mL	-

Table S3. Reagent stoichiometry for synthesis of DASA-functionalised microspheres. [b]Calculated assuming 100% conversion of VBC units to aniline

Polymer	Reagent	Quantity	mmol
**D1**	A1	0.26 g	0.25[b]
	Trifluoropyrazolone furan adduct (CF3PFA)	0.08 g	0.27
	Toluene	20 mL	-
**D2**	A2	0.25 g	0.66[b]
	Trifluoropyrazolone furan adduct (CF3PFA)	0.15 g	0.49
	Toluene	20 mL	-
**D3**	A3	0.25 g	0.91[b]
	Trifluoropyrazolone furan adduct (CF3PFA)	0.21 g	0.69
	Toluene	20 mL	-

Characterization methods

NMR spectroscopy was carried out at 298 K on a Bruker AV3 400 (400 MHz) spectrometer. The frequencies used were 400.13 MHz for ^{1}H nuclei, 100.61 MHz for ^{13}C nuclei. All chemical shifts (δ) are reported in parts per million (ppm) relative to tetramethylsilane with coupling constant (*J*) in Hz (multiplicity: s = singlet, d = doublet, dd = doublet of doublets, ddd = doublet of doublet of doublets, t = triplet, td = triplet of doublets, tt = triplet of triplets, q = quartet, quint. = quintet, m = multiplet, br = broad signal). Spectra were calibrated to the residual solvent peak of $CDCl_3$/DMSO-d_6.

FT-IR spectra were recorded on a Shimadzu IRAffinity-1 Spectrophotometer instrument.

High-resolution mass spectrometry (HRMS) was obtained from a Thermo-Fisher Scientific Exactive Plus Orbi-Trap Mass Spectrometer using electrospray ionisation

Experimental S1. Synthesis of *N*-phenylpropane-1,3-diamine (1)

Cu(I)Cl (0.13 g, 1.3 mmol), potassium hydroxide (1.38 g, 24.6 mmol), iodobenzene (1.40 mL, 12.5 mmol) and propane-1,3-diamine (3.10 mL, 37.1 mmol) were added to a glass sample vial equipped with a magnetic stirrer bar. The reaction mixture was left stirring for 8 h at a temperature of 0 ˚C before being diluted with DI water (50 mL) and extracted with dichloromethane (DCM; 2 x 50 mL). The organic layers were collected and dried over magnesium sulfate, filtered and concentrated under reduced pressure. The crude product was filtered through a silica gel column (DCM/methanol 2:1 with 5 % triethylamine as eluent) to give a yellow oil as the final product (1.24 g, 69 %).

^{1}H NMR (400 MHz, $CDCl_3$): δ 7.17 (tt, *J* = 7.2, 1.2 Hz, 2H, Ar-C-*H*), 6.69 (tt, *J* = 7.2, 1.2 Hz, 1H, Ar-C-*H*), 6.62 (dd, *J* = 8.4, 0.8 Hz, 2H, Ar-C-H), 3.20 (t, *J* = 6.8 Hz, 2H, Ar-NH*CH2*-), 2.85 (t, *J* = 6.4 Hz, 2H, -*CH2*NH2), 1.76 (quint, *J* = 6.8 Hz, 2H, CH2*CH2*CH2); ^{13}C NMR (100 MHz, $CDCl_3$) δ 148.4, 129.2, 117.1, 112.7, 42.0, 40.1, 32.7; FT-IR (ATR) 3281, 2928, 2858, 1601, 1501, 1474, 1431, 1317, 1258, 1178, 1153, 1120, 1097, 1072, 989, 866, 820, 746, 692 cm^{-1}; HRMS (ESI+) *m/z* 151.1230 (151.1230 calc'd for $C_9H_{15}N_2^+$ $[M]^+$).

Experimental S2. Synthesis of 2-phenyl-5-(trifluoromethyl)-2,4-dihydro-3*H*-pyrazol-3-one (2)

Phenylhydrazine (5.50 mL, 55.9 mmol) and ethyl-4,4,4-trifluoroacetoacetate (7.00 mL, 47.9 mmol) were added to glacial acetic acid (10 mL) in a round-bottomed flask equipped with a magnetic stirrer bar and condenser. The reaction vessel was refluxed overnight (18-20 h) before being allowed to cool to room temperature. The solid product was isolated by vacuum filtration through a Buchner funnel, washing with DI water and hexane to yield a yellow solid (5.33 g, 49 %).

^{1}H NMR (400 MHz, DMSO-d6): δ 7.70 (dd, 2H, J = 7.2 and 2.0 Hz, Ar-C-*H*), 7.51 (td, 2H, J = 7.6 and 2.0 Hz, Ar-C-*H*) 7.38 (tt, 1H, J = 7.6 and 1.2 Hz, Ar-C-*H*), 5.93 (s, 2H. -C(O)*CH2*-); FT-IR (ATR) 2675, 1683, 1608, 1568, 1498, 1429, 1402, 1313, 1255, 1232, 1157, 1136, 1099, 983, 860, 769, 717 cm $^{-1}$.

S6. Synthesis of (*E*)-4-(furan-2-ylmethylene)-2-phenyl-5-(trifluoromethyl)-2,4-dihydro-3*H*-pyrazol-3-one (CF3PFA; 3)

(2) (4.96 g, 21.7 mmol), furfural (15.00 mL, 181.1 mmol) and *L*-proline (0.31 g, 2.7 mmol) were added to a round-bottomed flask equipped with a magnetic stirrer bar. The reaction mixture was stirred overnight at room temperature to yield a dark red/black solution. The product was purified *via* flash silica column chromatography using hexane as the eluent to yield an orange solid (0.98 g, 15 %).

^{1}H NMR (400 MHz, $CDCl_3$): δ 8.93 (d, *J* = 4.0 Hz, 1H, furan-C*H*-), 7.92 (dd, *J* = 8.8, 1.2 Hz, 2H, Ar-C-*H*), 7.88 (d, *J* = 0.4 Hz, 1H, furan-C*H*-), 7.70 (s, 1H, C=C*H*-C(furan)), 7.46 (t, *J* = 8.0 Hz, 2H, Ar-C-*H*), 7.29 (tt, *J* = 7.2 Hz, 1H, Ar-C-*H*), 6.80 (ddd, *J* = 3.6, 1.6, 0.8 Hz, 1H, furan-C*H*-); ^{13}C NMR (100 MHz, $CDCl_3$) δ 161.4, 150.8, 150.6, 137.6, 131.5, 129.0, 127.9, 126.1, 121.8, 119.9, 119.4, 115.8, 115.6; FT-IR (ATR) 3146, 3115, 1697, 1595, 1531, 1497, 1448, 1433, 1356, 1335, 1319, 1211, 1180, 1111, 1095, 1028, 972, 934, 885, 806, 777, 752, 714, 690, 669 cm^{-1}; HRMS (ESI+) *m/z* 307.0682 (307.0689 calc'd for $C_{15}H_{10}O_2N_2F_3^{+}$, [M]$^{+}$).

SI Supplementary Data

Solid state NMR spectra & calculations for D1-3

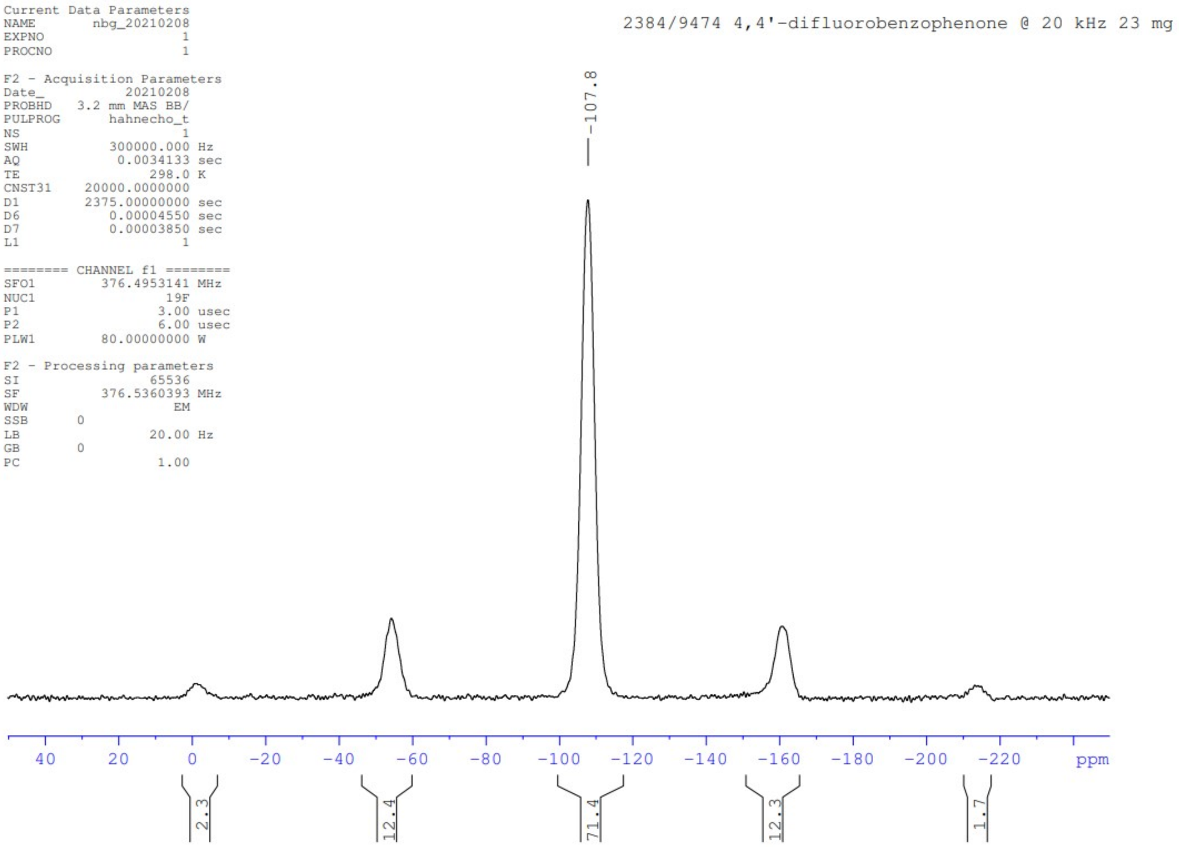

Figure S1: ^{19}F ssNMR spectrum of the external standard difluorobenzophenone (DFB).

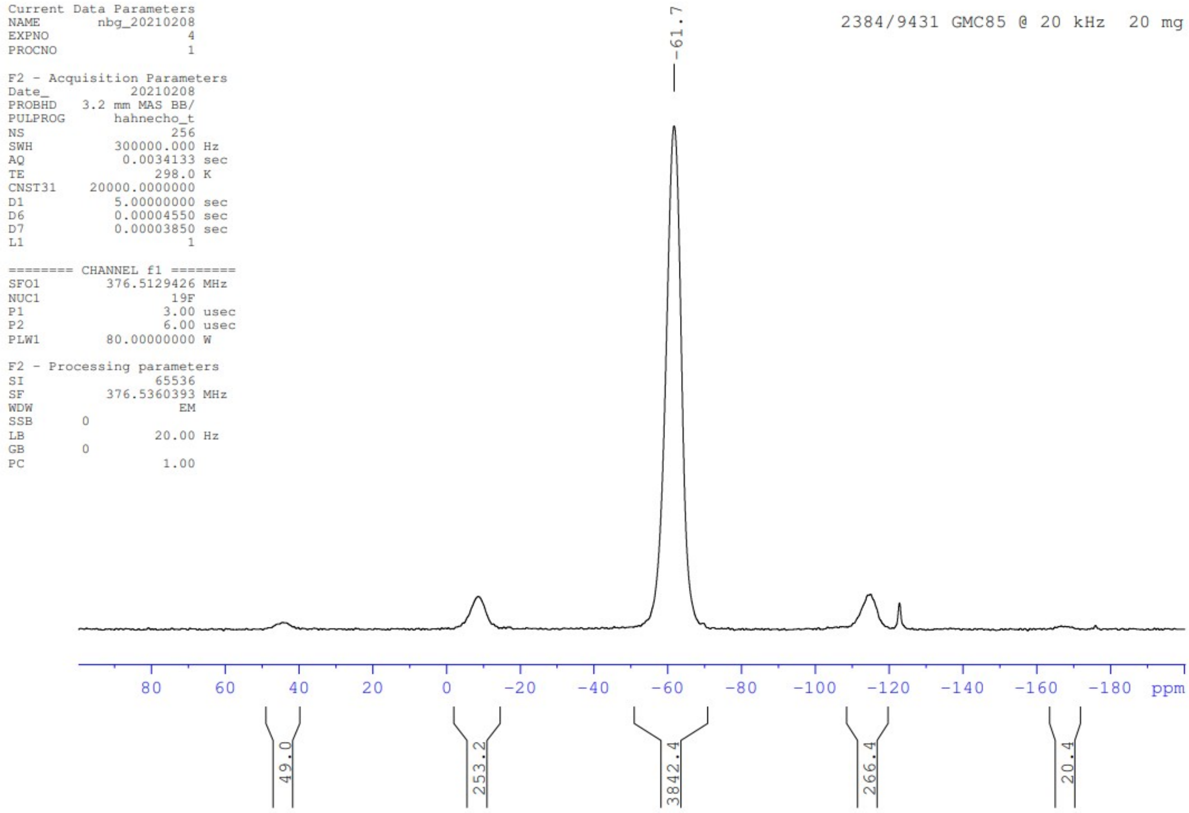

Figure S2. ^{19}F **ssNMR spectrum of D1.**

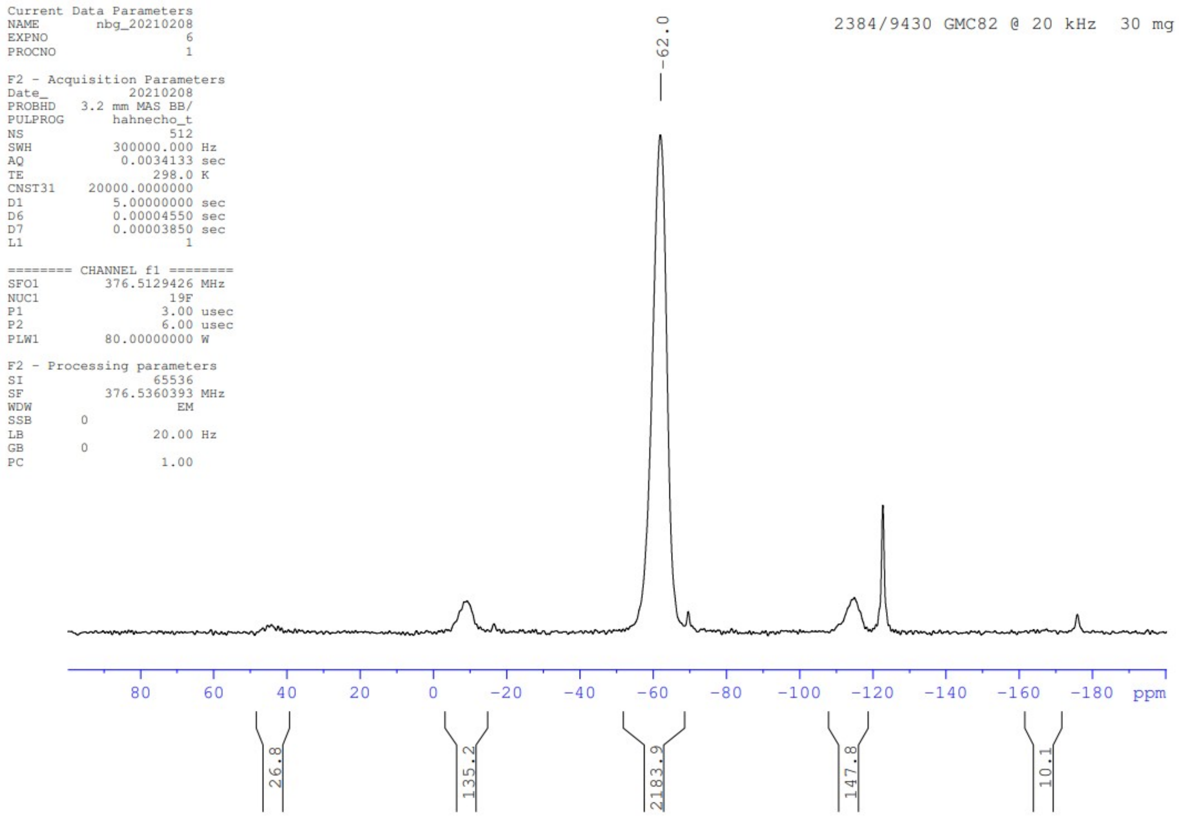

Figure S3. ^{19}F **ssNMR spectrum of D2.**

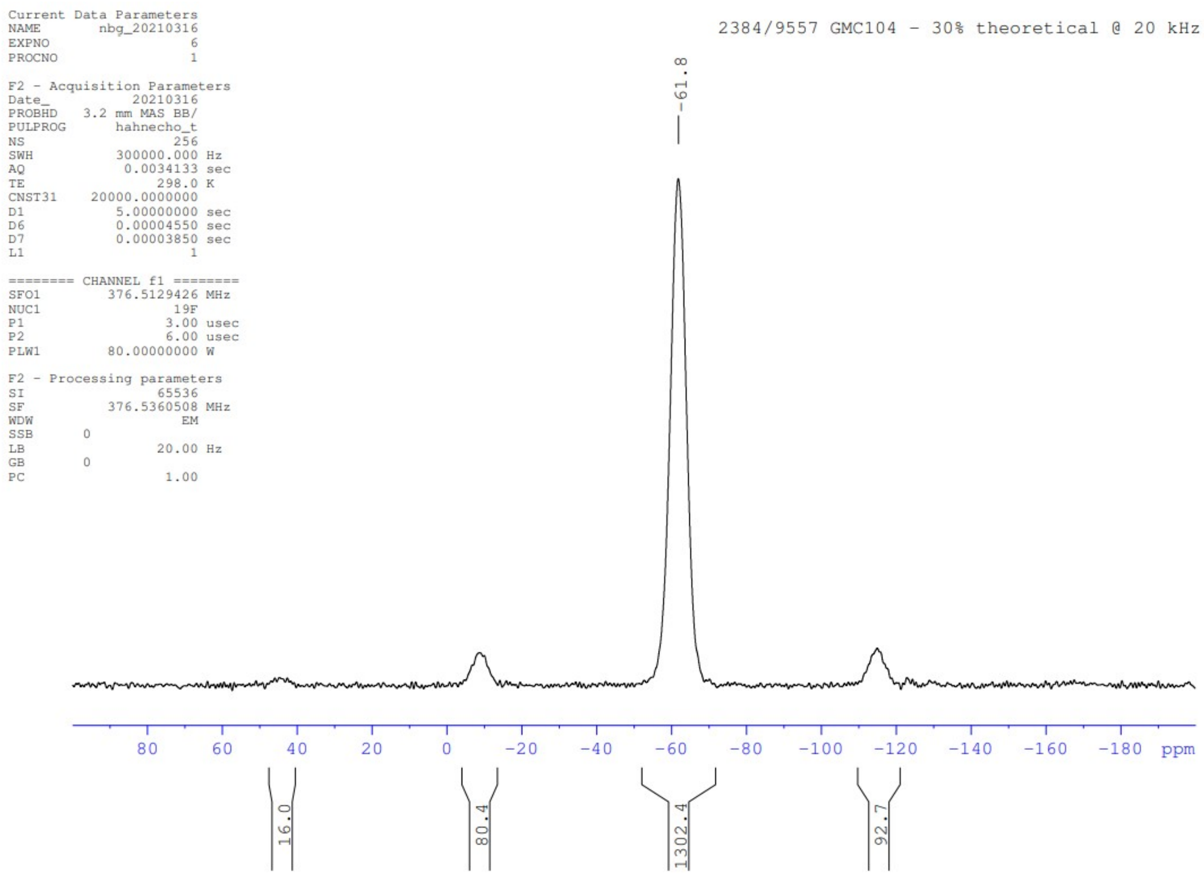

Figure S4. ^{19}F ssNMR spectrum of D3.

To determine the functionalisation degree of the microspheres from the normalised integral per mg / a.u. the following calculations were performed:

Due to the variance in fluorine T_1 relaxation and experimental repetitions for the DFB external standard (T_1 = 473 s) and the DMs (T_1 = 790 ms), raw integral values were corrected with respect to the number of repetitions and then normalised to get an integral per mg value.

Table S4. Adjustment of raw integral values considering number of repetitions and sample mass

Sample	Mass (mg)	Raw integral (a.u.)	Repetitions	Integral corrected for repetitions	Integral per mg	Normalised integral per mg (a.u.)
Standard	23	100	1	100	4.35	100
D1 (GMC82)	30	2504	512	4.89	0.163	3.75
D2 (GMC104)	22	1492	256	5.83	0.26	6.1
D3 (GMC85)	20	4431	256	17.3	0.865	19.9

The fluorine content per mole of DFB external standard was calculated:

$N(F)_i = N(F)_{molecule}N_A$

Where $N(F)_{molecule}$ is the number of fluorine atoms in the molecule and N_A is Avogadro's number

$$N(F)_{mol(DFB)} = 2 \times 6.022 \times 10^{23} = 1.204 \times 10^{24}$$

$N(F)_{mol(DFB)}$ represents the number of ^{19}F atoms in one mole of difluorobenzophenone.

The fluorine content represented by 1 normalised integral unit could then be calculated:

$$N(F)_{IU} = \frac{n_{DFB}N(F)_{mol(DFB)}}{I_N}$$

Where n_{DFB} is the moles of DFB used in the NMR experiment and I_N is the normalised integral value. Since integral values between standard and samples were normalised according 1 mg of substance, n_{DFB} is equal to $\left(\frac{1 \times 10^{-3} g}{218.2\ g/mol}\right)$ or 4.583 x 10^{-6} mol.

$$N(F)_{IU} = \frac{\left(\frac{1\times10^{-3}}{218.2}\right) \times N(F)_{mol(DFB)}}{100} = 5.518 \times 10^{16}$$

Where $N(F)_{IU}$ represents the number of fluorine atoms equal to one normalised integral unit, 218.2 g mol^{-1} is the molar mass of DFB.

Knowing the fluorine content represented by 1 normalised integral unit, the functionalisation degree of the microspheres could be calculated (example below for **D3**, 50 mol% VBC in initial monomer feed, 25 mol% aniline functionalisation).

Experimental fluorine content as observed by ssNMR spectroscopy at -61.7 ppm for **D3**:

$N(F)_{D3} = IU \times N(F)_{IU} = 1.098 \times 10^{18}$

The theoretical maximum fluorine content per mg microsphere (**D3**) was calculated based off the available aniline groups, i.e. the degree of aniline functionalised determined from elemental analysis.

$$N(F)_{D3(theo)} = \left(\frac{m}{M_{w(sphere)}}\right)3N_A f_M = \left(\frac{1 \times 10^{-3}}{244.28}\right)3N_A \times 0.25 = 1.849 \times 10^{18}$$

$M_{w(sphere)}$ is the weighted average molecular weight of the microsphere, according to the initial monomer feed, conversion to chlorine groups to aniline groups (determined from elemental analysis) and assuming all aniline groups react to form DASA groups:

$$\sum_{i=1}^{n} M_i \varphi_i$$

Where M_i is the molecular weight of a monomer species and φ_i is its mole fraction.

f_M is the maximum functionalisation degree (in this case 25 mol%). N_A is Avogadro's constant. Multiplying by a factor of 3 was done to represent the 3 fluorines of the CF_3 on the DASA units.

The conversion of the aniline units to the DASA is then represented by:

$$\frac{N(F)_{D3exp}}{N(F)_{DEtheo}} \times 100\% = 59\%$$

The overall degree of DASA functionalisation for the microspheres is finally determined by considering the aniline conversion with respect to the initial aniline functionalisation degree:

$$\left(\frac{N(F)_{D3}}{N(F)_{D3(theo)}}\right) f_{M\%} = 14.8\, mol\%\ DASA\ functionalised\ microsphere$$

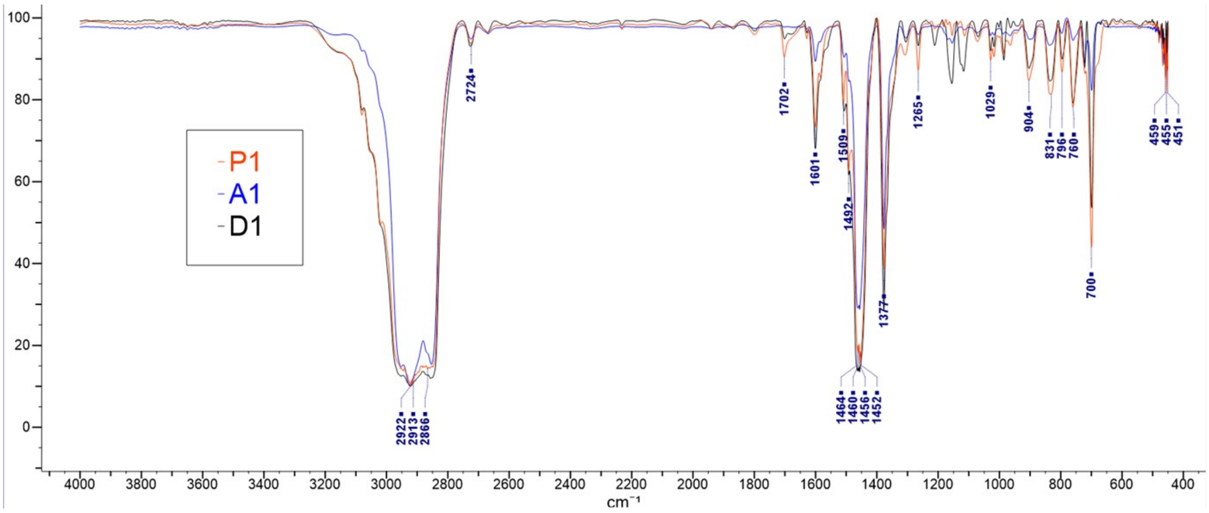

Figure S5. FT-IR Spectra of P1, A1, and D1.

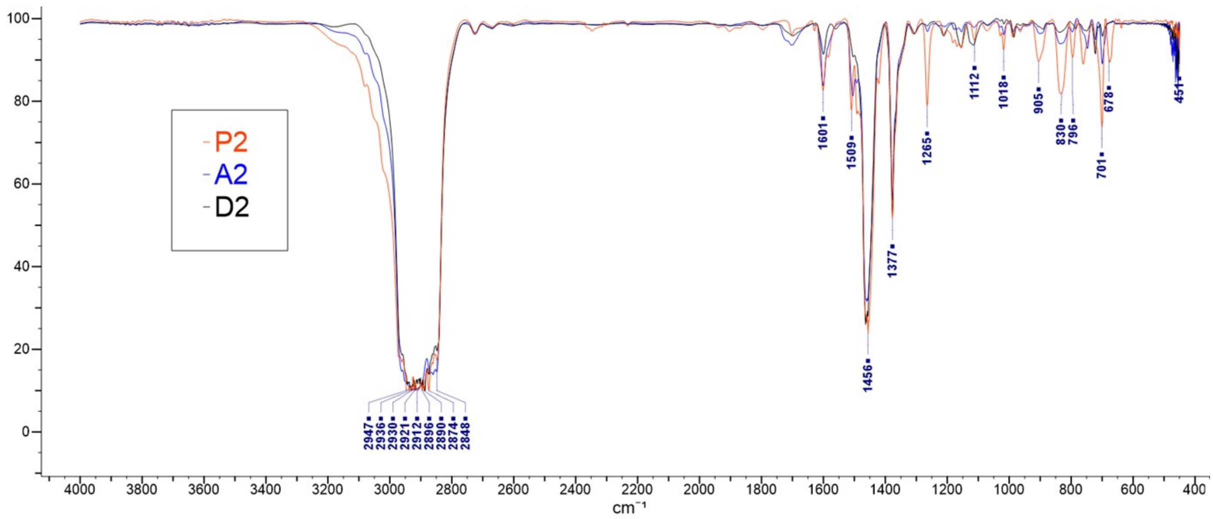

Figure S6. FT-IR Spectra of P2, A2, and D2.

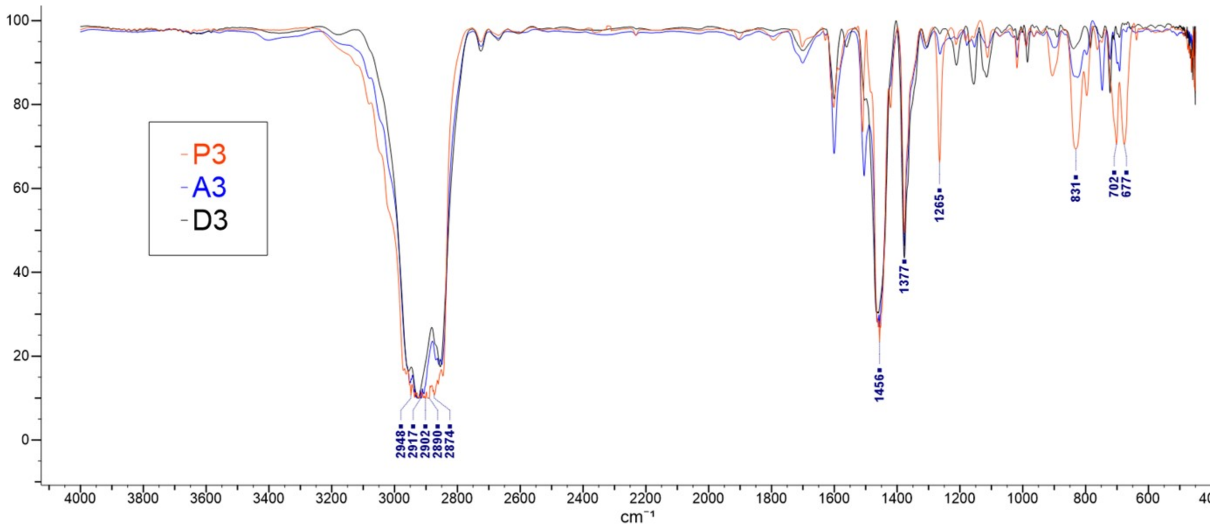

Figure S7. FT-IR Spectra of P3, A3, and D3.

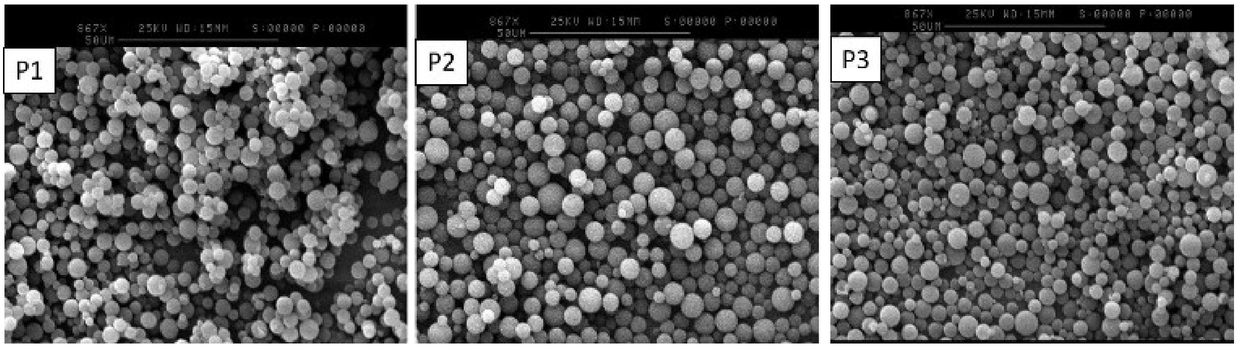

Figure S8. SEM Images of P1-3, scale bars = 50 μm

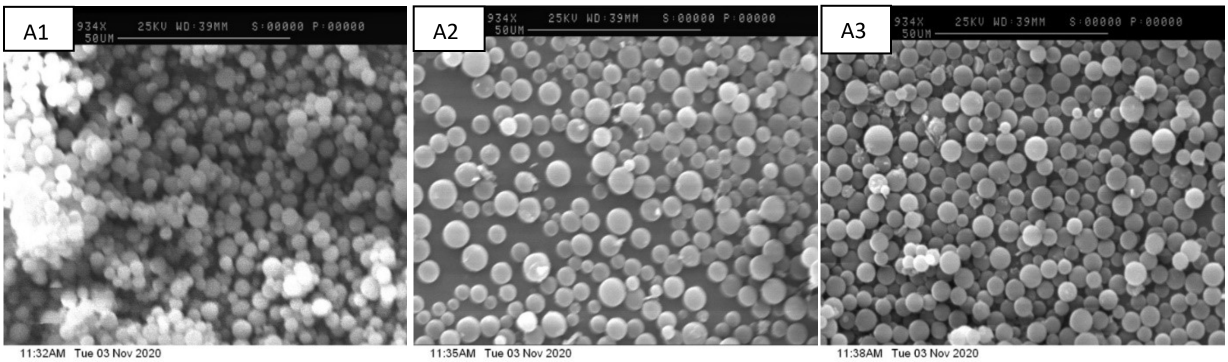

Figure S9. SEM Images of A1-3, scale bars = 50 µm.

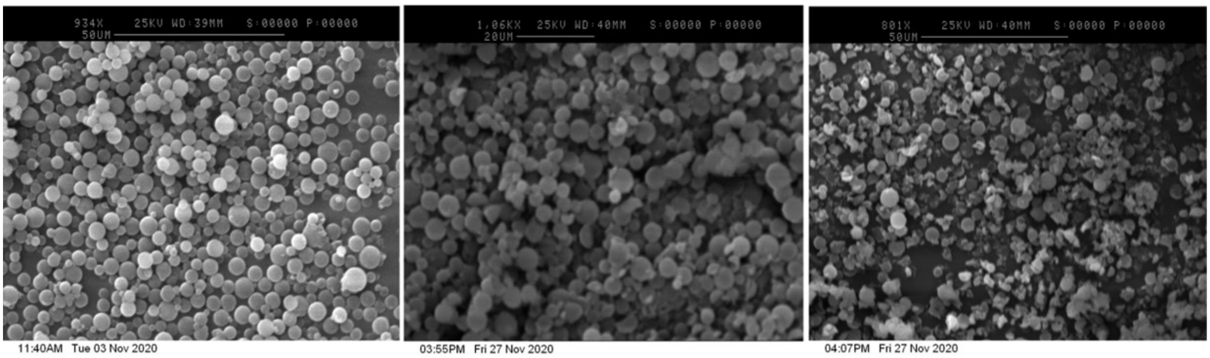

D1 in toluene; Initial, 4 hour irradiation, 24 hour irradiation

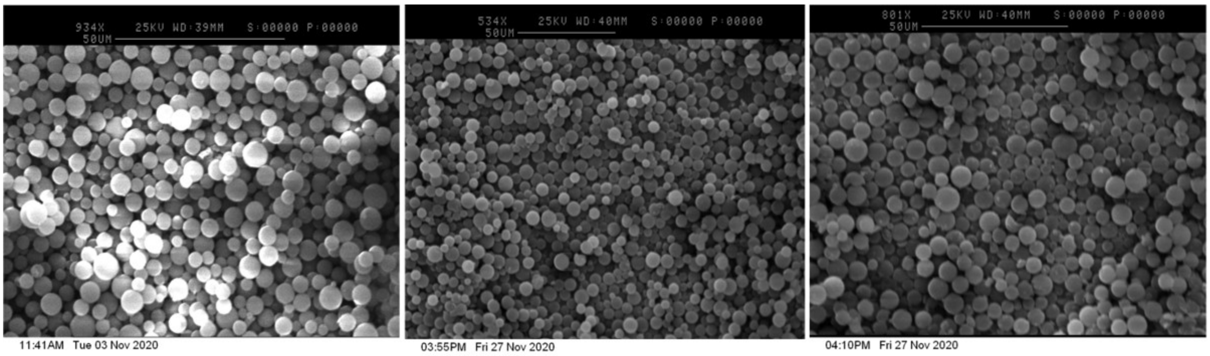

D2 in toluene; Initial, 4 hour irradiation, 24 hour irradiation

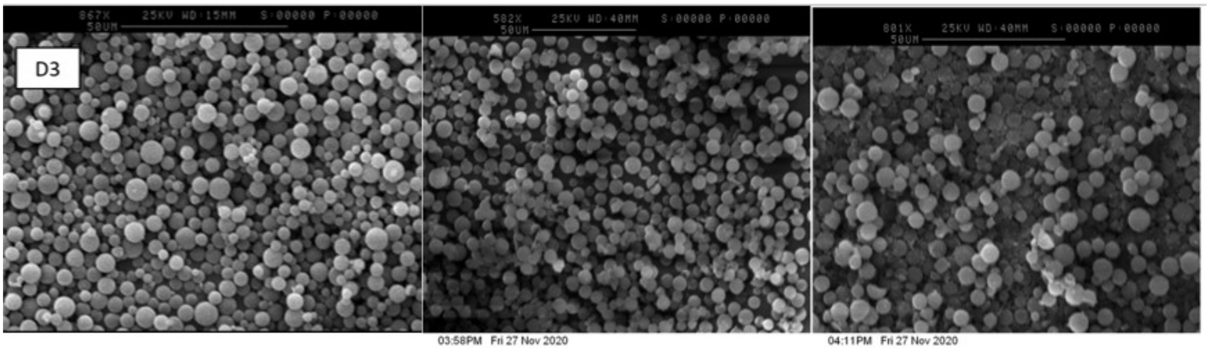

D3 in toluene; Initial, 4 hour irradiation, 24 hour irradiation

Figure S10. SEM Images for D1-3 after irradiation with white light for 0 h, 4 h, and 24 h, Scale bars = 50 μm.

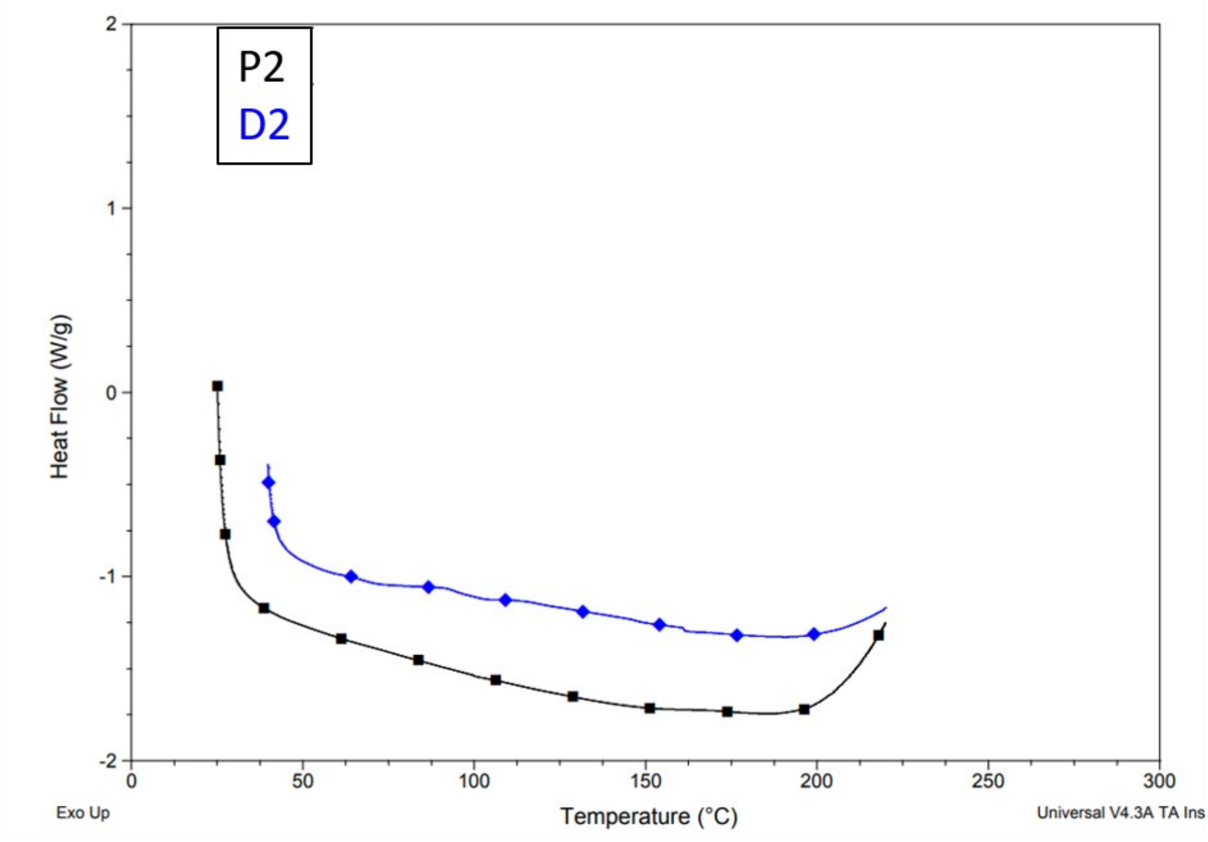

Figure S11. DSC curves of D2.

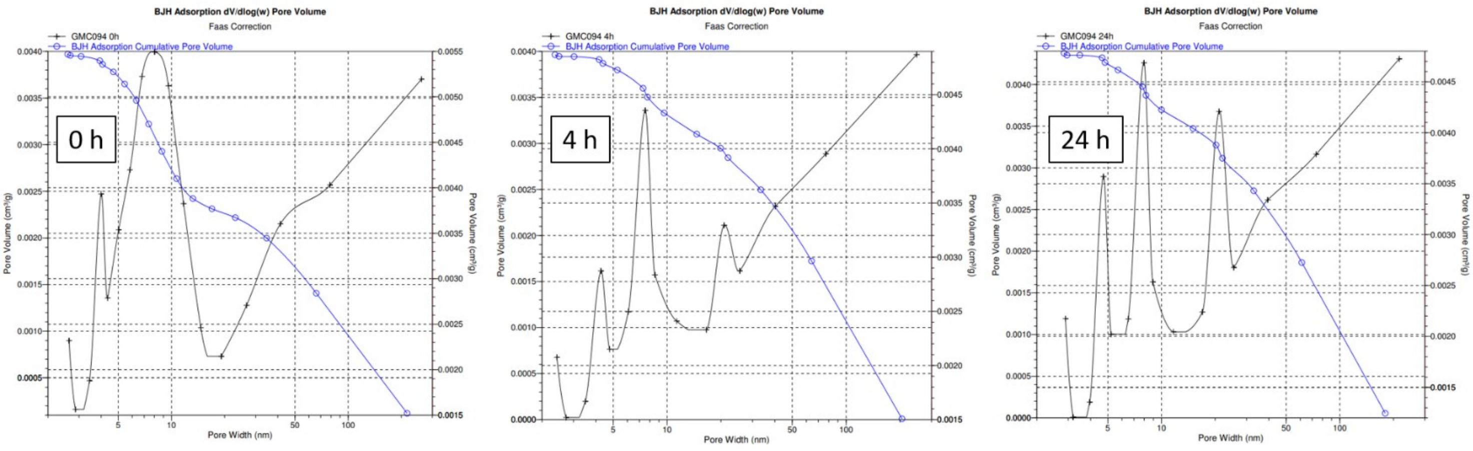

Figure S12. Barret-Joyner-Halenda pore volume distribution for D2 after various lengths of white light irradiation in toluene obtained *via* nitrogen sorption analysis.

Raw UV-Vis data from integrated sphere spectrophotometer

Raw data processing: The raw data was processed using Origin software package. The raw data was smoothed using the Savitzky-Golay method. To account for baseline drifts due to the heterogeneous dispersion, absorbance values of spectra were corrected at 700 nm before being normalised.

From left to right: D1/2/3 UV-Vis spectra during irradiation with ThorLabs 630 nm LED

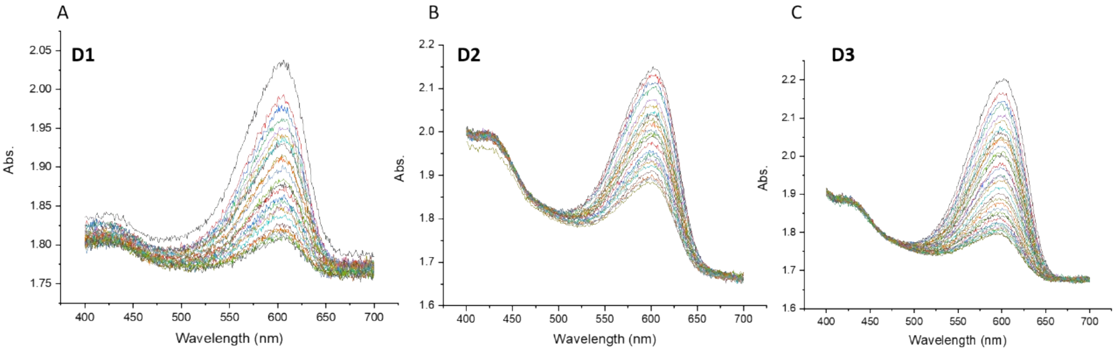

array

Figure S13. UV-Vis spectra of a) D1, b) D2, and c) D3 during irradiation with ThorLabs

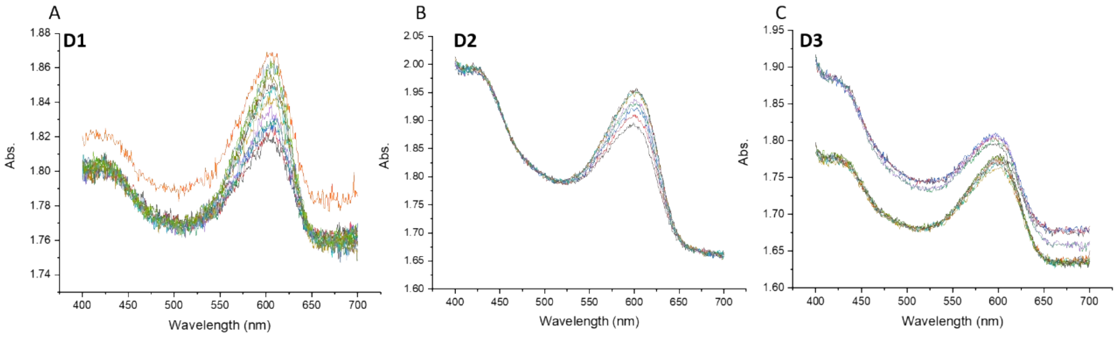

630 nm LED array (raw UV-Vis data from integrated sphere spectrophotometer).

Figure S14. UV-Vis spectra of a) D1, b) D2, and c) D3 during thermal equilibration in the dark (raw UV-Vis data from integrated sphere spectrophotometer).

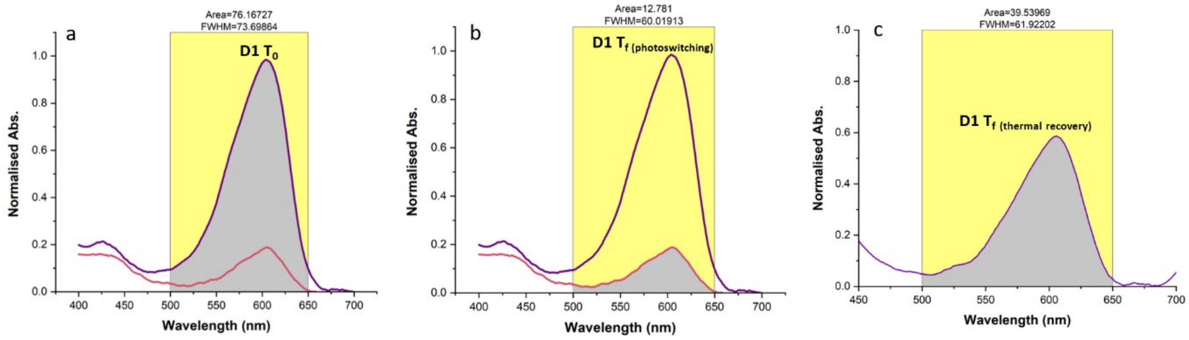

D1 – T_0, forward photoswitching T_f, thermal recovery T_f

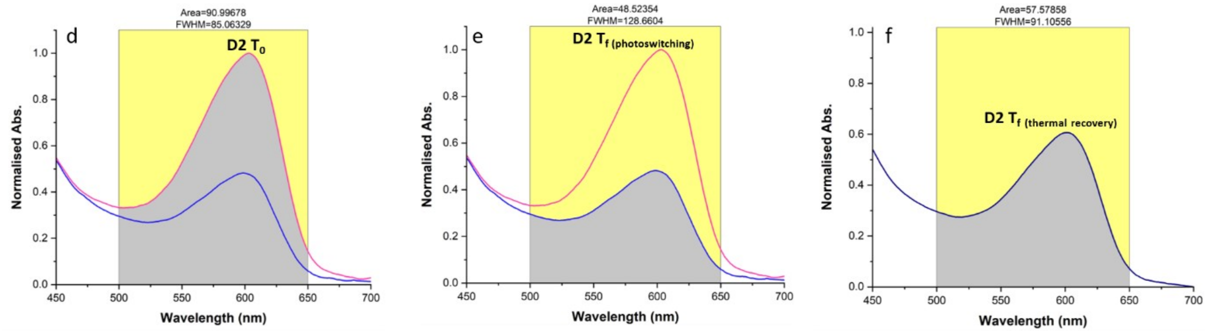

D2 – T_0, forward photoswitching T_f, thermal recovery T_f

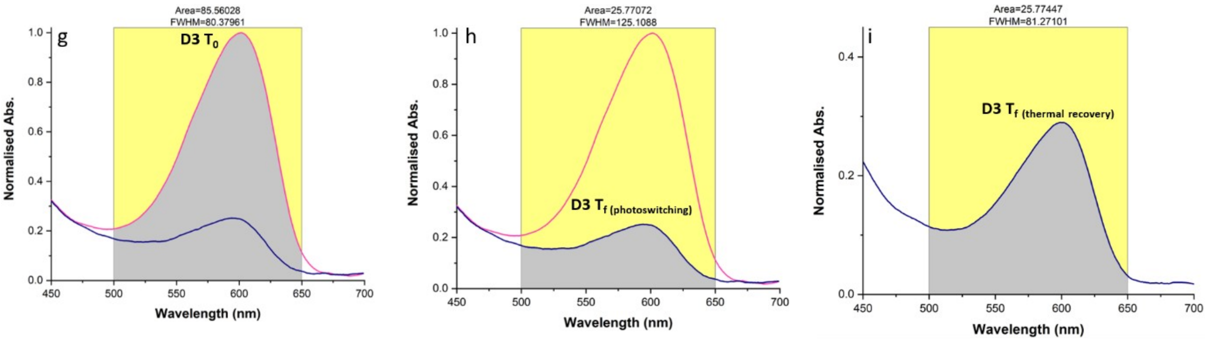

D3 – T_0, forward photoswitching T_f, thermal recovery T_f

Figure S15. Integration of DASA absorbance bands of a-c) D1, d-f) D2, and g-i) D3 to measure conversion during white light irradiation and thermal recovery as reported in Figure 4 j-l.
